# Adipogenic and SWAT cells separate from a common progenitor in human brown and white adipose depots

**DOI:** 10.1038/s42255-023-00820-z

**Published:** 2023-06-19

**Authors:** Nagendra P. Palani, Carla Horvath, Pascal N. Timshel, Pytrik Folkertsma, Alexander G. B. Grønning, Tora I. Henriksen, Lone Peijs, Verena H. Jensen, Wenfei Sun, Naja Z. Jespersen, Christian Wolfrum, Tune H. Pers, Søren Nielsen, Camilla Scheele

**Affiliations:** 1 Novo Nordisk Foundation Center for Basic Metabolic Research, University of Copenhagen, Copenhagen, Denmark; 2 ZS Associates, Copenhagen, Denmark; 3 The Center of Inflammation and Metabolism and the Center for Physical Activity Research, Rigshospitalet, University of Copenhagen, Copenhagen, Denmark; 4 Institute of Food, Nutrition and Health, ETH Zurich, Zurich, Switzerland; 5 The Novo Nordisk Foundation Center for Genomic Mechanisms of Disease, Broad Institute of MIT and Harvard, Cambridge, MA USA

**Keywords:** Mesenchymal stem cells, Fat metabolism

## Abstract

Adipocyte function is a major determinant of metabolic disease, warranting investigations of regulating mechanisms. We show at single-cell resolution that progenitor cells from four human brown and white adipose depots separate into two main cell fates, an adipogenic and a structural branch, developing from a common progenitor. The adipogenic gene signature contains mitochondrial activity genes, and associates with genome-wide association study traits for fat distribution. Based on an extracellular matrix and developmental gene signature, we name the structural branch of cells structural Wnt-regulated adipose tissue-resident (SWAT) cells. When stripped from adipogenic cells, SWAT cells display a multipotent phenotype by reverting towards progenitor state or differentiating into new adipogenic cells, dependent on media. Label transfer algorithms recapitulate the cell types in human adipose tissue datasets. In conclusion, we provide a differentiation map of human adipocytes and define the multipotent SWAT cell, providing a new perspective on adipose tissue regulation.

## Abstract (web summary)

Single-cell transcriptomic analysis of progenitor cells from human adipose depots reveals an adipogenic and a structural branch of cells, the latter named SWAT cells and shown to display a multipotent phenotype.

## Main

Body fat distribution and adipocyte functionality are determinants of metabolic health in a depot-dependent fashion. Multiple studies have described that abdominal obesity is strongly associated with cardiovascular disease and insulin resistance, whereas accumulation of fat in the lower gynoid regions has a lower lipid turnover and is associated with metabolic health^1^. Brown adipose tissue (BAT) activity is also associated with metabolic health^2^. Adipocytes originate from mesenchymal stem cells that reside in multiple tissues including adipose tissue, skeletal muscle and bone marrow^3^. Other studies have shown a close relation between osteocytes and adipocytes, with opposing differentiation trajectories mediated through a common transcriptional network^4^. Intriguingly, progenitor cells derived from the seasonally plastic white adipose tissue (WAT) of brown bears spontaneously differentiated into osteocytes in vitro^5^, further emphasizing the developmental link between these two cell types.

Single-cell technologies have allowed an understanding of the heterogeneity of adipocytes, revealing several subtypes with specialized functions^6–8^. These studies suggest that separate cell types provide thermogenesis, insulin sensitivity, lipid storage and adipokine secretion^9–11^ or act as negative regulators of lipid accumulation^12^. However, the developmental hierarchical heterogeneity of human brown and white adipogenic events remains elusive. In the current study, we compared human adipose stem and progenitor cells (hASPCs) derived from two WAT depots—subcutaneous and visceral—and from two BAT depots—supraclavicular and perirenal. We study these cells during early differentiation and combine computational modelling with experimental cell separation techniques to describe two cell-type branches that arise from a common progenitor. Finally, we confirm that all these cell types are present in vivo using machine learning-based label transfer algorithms. Gene expression plots for our data can be visualized using the web tool available at: https://cphbat.shinyapps.io/adipodiff/.

## Results

### Human white and brown adipose tissue progenitors share early cell fates

**Fig. 1:**
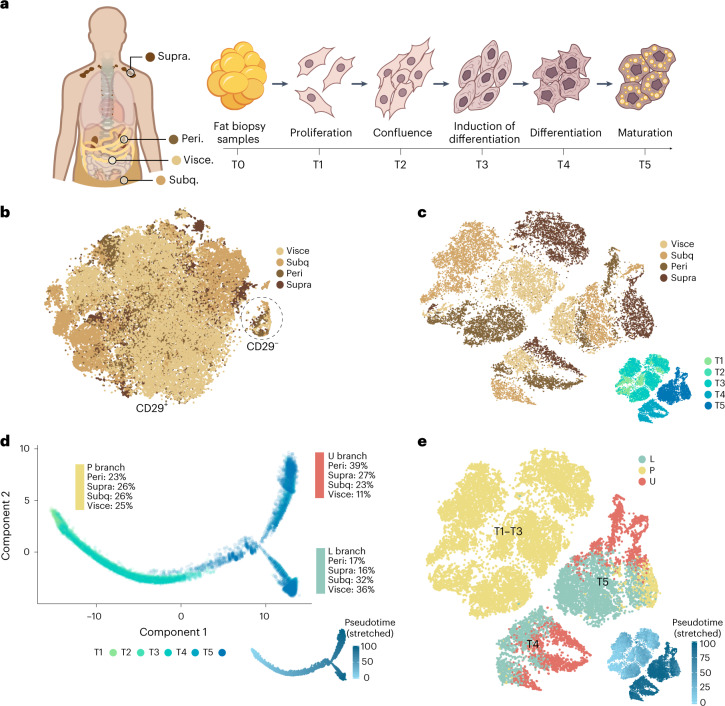
Single-cell trajectory analysis of developing adipocyte progenitors. Human adipocyte progenitors isolated from tissue biopsies of four adipose depots were collected at five time points (T1–T5) during in vitro differentiation and subsequent single-cell analysis was performed using the 10x Genomics platform. **a**, Overview of adipose depots and cellular developmental stages. **b**, *t*-SNE atlas generated using the Seurat alignment algorithm, analysing proliferating adipocyte progenitors (T1) derived from 11 individuals. Peri, perirenal; subq, subcutaneous; supra, supraclavicular; visce, visceral. **c**, *t*-SNE atlas of developing adipocyte progenitors (T1–T5) of four adipose depots from different individuals. The inset indicates the collection time point. Clustering analysis grouped T1–T3 samples by adipose depot and T4 and T5 samples by time point and adipose depot. **d**, Monocle pseudotime trajectory of adipocyte progenitors from T1–T5. Cells from T1–T3 align in a common progenitor (P) branch. Following induction of differentiation, the P branch split into an upper (U) and a lower (L) branch, thus containing cells from T4 and T5. Cells from all depots are represented in the U and L branches (the percentage of cells from each depot is indicated for each branch). The inset shows the trajectory coloured by stretched pseudotime that quantitatively measures how far an adipocyte progenitor has progressed through development. Stretched pseudotime is a normalized pseudotime scale ranging from 0 (least progressed) to 100 (most progressed). **e**, Cell atlas coloured by trajectory branch identities. The inset shows the cellular development as measured by pseudotime.

**Extended Data Fig. 1:**
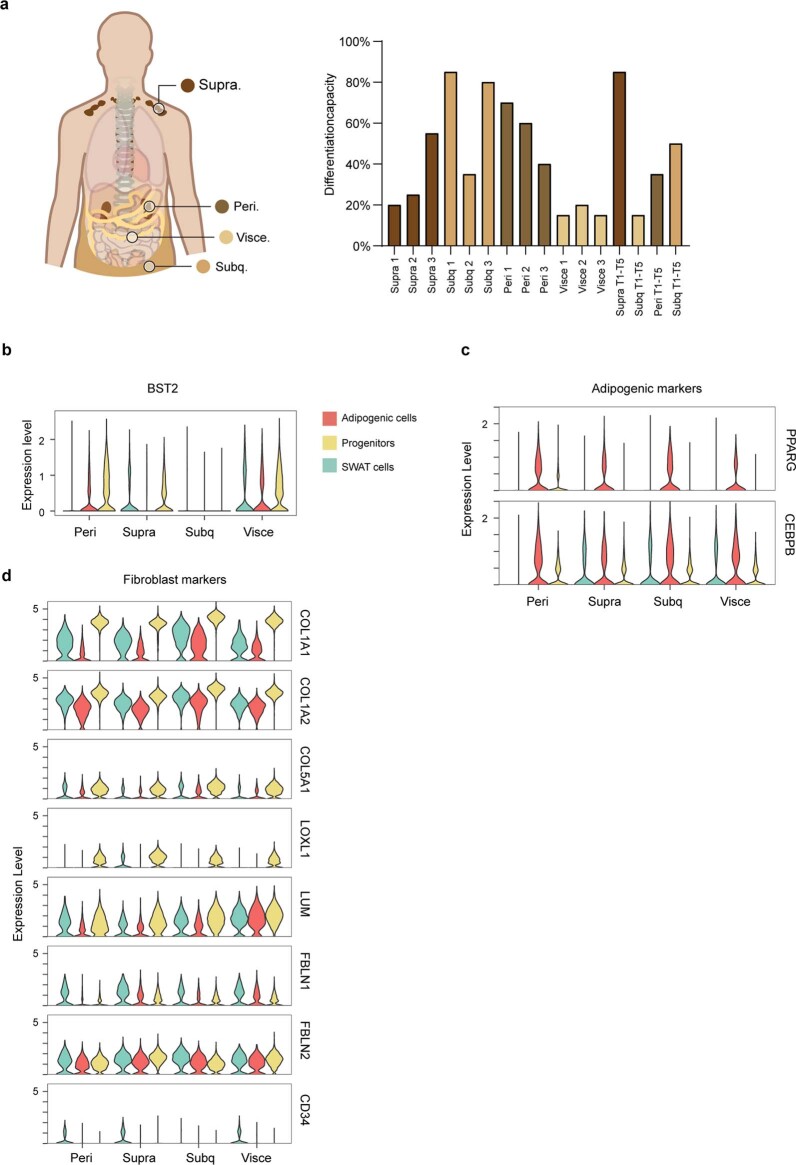
Characteristics of the cell cultures included in the scRNA seq experiments. **a)** Differentiation capacity assessed by visual estimation of percentage of accumulated lipid droplets. **b)** Violin plots of *BST2* gene expression divided among branches and based on depot origin. **c)** Violin plots of the adipogenic markers *PPARG* and *CEBPB* gene expression divided among branches and based on depot origin. **d)** Violin plots of multiple fibroblast markers gene expression divided among branches and based on depot origin.

Human adipocytes differ between depots. For example, subcutaneous adipocytes have been found to be more efficient in storing lipids than visceral adipocytes^13^, whereas supraclavicular and perirenal adipocytes display a heterogeneous composition of both thermogenic multilocular adipocytes and more white-like unilocular adipocytes^14,15^. These differences are to some extent reflected in isolated progenitors that are differentiated in vitro^14–16^. To investigate cellular differences in adipose depots, we cultured 14 different adipose progenitor cell strains derived from supraclavicular or perirenal BAT or from visceral or subcutaneous WAT of adult humans^14,15^ (Fig. 1a, Extended Data Fig. 1a and Supplementary Table 1). We generated droplet-based single-cell RNA-sequencing (scRNA-seq) data from these adipose progenitor cell strains and obtained 56,371 high-quality cells after quality control.

For the initial analysis, we collected cells at a proliferating, sub-confluent state (‘T1’; Fig. 1a). For each depot, we included cells from at least three different individuals, matched for age, sex and body mass index (BMI; Supplementary Table 1). We explored initial clustering by performing regression analyses (Supplementary Fig. 1) and by aligning the samples to each other using canonical correlation analysis and dynamic time warping (Supplementary Fig. 2). These analyses suggest that both cell cycle effects and batch effects were driving the clustering of the data. This was further visualized using two approaches for organizing single-cell data according to RNA expression (Monocle^17^) or splicing patterns (Velocyto^18^; Supplementary Fig. 3).

When adjusting for batch and cell cycle effects, our data suggested that undifferentiated, proliferating adipocyte progenitors have similar expression patterns regardless of BAT or WAT origin (Fig. 1b). The observed similarity between human BAT-derived and WAT-derived adipocytes was somewhat surprising but possibly related to the fact that genes unrelated to adipocyte function were dominating at this developmental stage. We analysed the data further and did, however, identify a small number of depot-selective genes (Supplementary Note, Supplementary Table 2 and Supplementary Fig. 4).

We found that most progenitors grouped into a *CD29*-positive cluster, whereas a smaller cluster with *CD29*-negative cells was also formed. Cells from all four depots contributed to both clusters (Fig. 1b). CD29 is a surface marker previously proposed to be a predictive marker for adipogenic precursor cells with thermogenic potential^19^. We characterized the two clusters by using scmap^20^ to map our dataset onto an existing catalogue of cell types in murine tissues^21^. In this analysis, the large *CD29*-positive cluster was enriched for adipogenic precursor markers, whereas the smaller *CD29*-negative cluster was enriched for epithelial cell markers (Supplementary Fig. 5).

Most genes distinguishing between brown and white adipocytes are not expressed until later in differentiation^22^. We therefore next harvested cells from all four depots during four additional time points, T2–T5, during differentiation (Fig. 1a and Supplementary Table 3) and performed scRNA-seq, obtaining 23,428 high-quality cells (Supplementary Fig. 6). At T3, cells were 2 d after confluence and a differentiation initiation cocktail was added. The same differentiation components were added to cells regardless of depot. Cells were subsequently harvested at T4 and T5, representing 3 and 6 days following addition of the differentiation cocktail, respectively. The full differentiation protocol into mature adipocytes is 12 d from the addition of the differentiation medium^23^. However, changes in cell morphology are initiated shortly after adding the cell differentiation medium, and at T5 (corresponding to 6 d after adding the differentiation medium), some accumulation of small lipid droplets has started. The analysis resulted in clearly separated cell clusters both by time point and by depot within T4 and T5, whereas a principal-component analysis (PCA) plot of the data shows that cells from T1–T3 overlap (Fig. 1c and Supplementary Fig. 6).

To assess common or distinct developmental trajectories across depots, we used Monocle^17^ to order cells in ‘pseudotime’. This analysis allows for a quantitative measure of progress through a biological process (Fig. 1d). We applied a data-driven approach without including prior information on adipose depot origin. The trajectory topology was robust to changes in the input and parameter settings of the Monocle trajectory algorithm (Supplementary Fig. 7). The trajectory analysis revealed bifurcating cell fates of adipose progenitor cells from the four different depots of human BAT and WAT. Cells from T1–T3 formed a progenitor (P) branch. In line with our initial analysis of proliferating progenitor cells, cells from the earlier time points (T1–T3) did not separate in pseudotime and cells from all depots contributed equally to the P branch (Fig. 1d). Later in pseudotime, cells separated into two branches: upper (U) branch and lower (L) branch containing cells after induction of differentiation (T4 and T5; Fig. 1d).

Following branching, we observed a depot-dependent asymmetry in cell distribution where the U branch was dominated by cells from the brown fat depots (>60%), while cells from the white fat depots (>60%) were overrepresented in the L branch. To address whether the branch division related to a thermogenic versus non-thermogenic signature, we plotted the expression of *BST2*, a recently identified marker for thermogenic progenitor cells in murine tissue^24^. Strikingly, in our human dataset, *BST2* was expressed in the progenitors derived from the two brown fat depots and the visceral adipose depot but not in the subcutaneous depot (Extended Data Fig. 1b). All three depots expressed *BST2* in the progenitors as well as in upper and lower branches between depots, to a varying extent (Extended Data Fig. 1b). Considering that thermogenic cells have been detected in visceral, but not subcutaneous adipose tissue in humans^9,25^, these data support that *BST2* might be a marker for thermogenic progenitors also in humans. We next plotted the adipogenic markers *PPARG* and *CEBPB* (Extended Data Fig. 1c), demonstrating a strong bias towards expression in the U branch cells. We also plotted a range of fibroblast markers (Extended Data Fig. 1d). These markers had variable bias towards highest expression in either progenitor cells or L branch cells, with some variation between depot origins. Importantly, all four depots contributed to both upper and lower cell branches (Fig. 1d). When *t*-distributed stochastic neighbour embedding (*t*-SNE) plots of cell branch identity were overlaid with time point and depot label, the branch separation occurring at both T4 and T5 was clear (Fig. 1e). Interestingly, a subpopulation of cells at T5, dominated by cells derived from the supraclavicular depot but including cells from all depots, was assigned to the P branch (Supplementary Table 4). These cells were only sporadically expressed at T4 and clustered in the late part of the P branch in pseudotime, suggesting that dedifferentiation had occurred (Supplementary Fig. 7). The mechanism of dedifferentiation of adipocytes has been previously reported^26^, possibly reflecting the ability of the cells to interconvert^27^. Supporting that dedifferentiation had occurred, differentially expressed genes between the T5 P branch cells and the T1–T3 P branch cells revealed residual high expression of both U branch-specific and L branch-specific markers, suggesting the unbiased contribution of dedifferentiated cells and gradual loss of branch-specific markers. These findings also emphasize that neither of the cells in the U branch or L branch are undifferentiated or dedifferentiated progenitor cells, but rather represent two separate cell fates present among differentiating adipocytes. Importantly, we were able to confirm the differentiation trajectories predicted by Monocle pseudotime using an independent method called Velocyto^18^ (Supplementary Fig. 8).

### A branching of adipogenic and SWAT cell fates in pseudotime

**Fig. 2:**
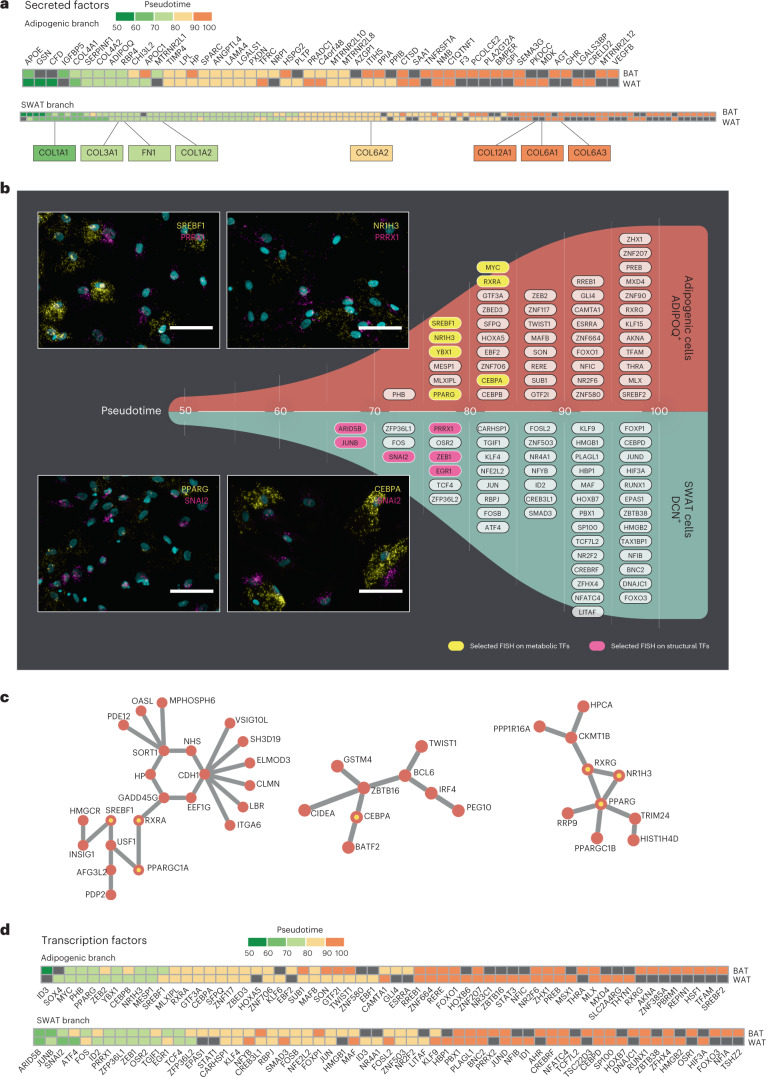
Adipocyte progenitors develop into adipogenic and SWAT cells. **a**, Adipogenic and SWAT branch-specific expression patterns of predicted secreted proteins, separated by origin from brown (supraclavicular + perirenal) and white (subcutaneous + visceral) depots. The SWAT branch is characterized by branch-specific expression of extracellular matrix components. The black box indicates no divergence in gene expression between branches. **b**, Transcription factors identified as increasing in expression in one branch over another are indicated on respective branches, at the stretched pseudotime point where the smoothed gene expression is observed to diverge. Inset images are from RNA FISH labelling in brown adipocytes of selected combinations of branch-specific transcription factors. Scale bar, 75 µm. **c**, Scellnetor analysis identified key transcriptional networks enriched in the adipogenic branch, confirming the role of several transcription factors identified above as involved in adipogenic cell-type development. **d**, Branch-specific transcription factor analysis performed independently for brown (perirenal + supraclavicular) and white (visceral + subcutaneous) depots. Colour scale indicates pseudotime point at which expression of a gene diverges between branches, and the black box indicates no divergence in gene expression between branches. A shared set of transcription factors characterize early differentiation across depots, whereas further differentiation proceeds through depot-specific transcription factors. TF, transcription factor.

To determine what defines the branches, we first predicted their cell-type-specific secretomes and ordered these in pseudotime. We utilized the published human secretome^28^ as a scaffold to predict the secreted products from the branch-specific genes. We performed separate analyses for cells derived from BAT (supraclavicular and perirenal) and WAT (subcutaneous and visceral), and we compared the pseudotime windows at which transcription factors diverged in expression between the branches. The U branch cells expressed well-established adipocyte derived factors including *ADIPOQ*, *LPL* and *SPARC* (Fig. 2a). The L branch cells encoded multiple extracellular matrix factors annotated as secreted. These included several types of collagens and fibronectin, proteins that are highly abundant in the development of obesity-induced fibrosis^29^ (Fig. 2a). The branch also expressed connective tissue growth factors including WNT1-inducible-signalling pathway protein 2 (WISP-2; Supplementary Table 5). The secretory capacity of the L branch cells was underscored by an approximately twofold increase in the number of genes encoding secreted factors compared to the U branch cells. (Fig. 2a).

We next explored the landscape of transcription factors in the dataset, identifying transcription factors with increasing branch-specific expression over pseudotime as drivers of branch development. Consistent with the U branch-specific expression of *ADIPOQ*, this branch was defined by peroxisome proliferator-activated receptor gamma (encoded by *PPARG*) and CCAAT/enhancer-binding protein alpha (encoded by *CEBPA*). These transcriptional activators synergistically activate adipogenesis and control genes important for adipocyte metabolism^30^ (Fig. 2b). Additional transcription factors selective for the U branch were *SREBF1* and *NR1H3*, which are stimulators of lipogenesis^31,32^. Based on the distinct secretion and transcription factor gene expression patterns, we defined the U branch cells as adipogenic cells. Intriguingly, the L branch cells induced a transcriptional programme driving osteogenic proliferation and differentiation. This programme included *SNAI2* and *JUNB*, both promoting osteoblast maturation and forming a transcription factor network described to compete with the adipogenic transcription factor network in mesenchymal stem cells^4^. Wnt signalling induces osteoblast differentiation in mesenchymal stem cells by suppressing PPAR-γ^33^, and we observed the Wnt signalling transcription factor, TCF7L2, also defining the L branch.

**Extended Data Fig. 2:**
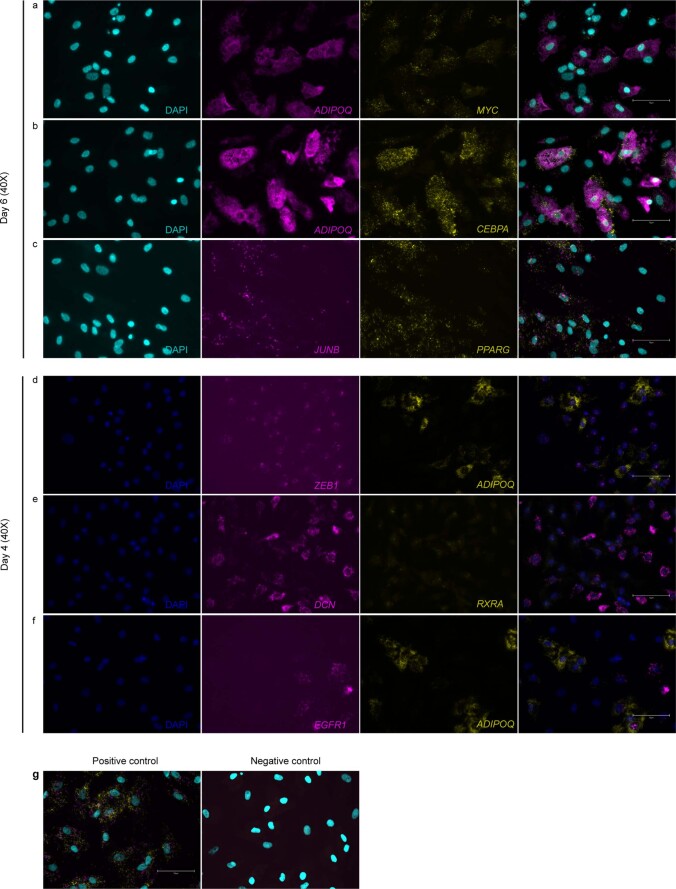
Fluorescent in Situ Hybridization (FISH) of transcription factors branching in pseudotime. Labelling of early timepoint mRNA markers defining the SWAT and adipogenic branch as annotated in Fig. 2. **a)** *ADIPOQ* (magenta) and *MYC* (yellow), representing the adipogenic branch, are co-localized in the same cell type. **b)** *ADIPOQ* (magenta) and *CEBPA* (yellow), representing the adipogenic branch, co-localized in the same cell type at day 6. **c)** *JUNB* (magenta) and *CEBPA* (yellow), represents the SWAT and adipogenic branch, respectively. FISH of the transcripts showed co-localization in the same cell type at day 6. **d)** *ZEB1* (magenta) and *ADIPOQ* (yellow), represents the SWAT and adipogenic branch, respectively. FISH analysis supported the singe cell data showing expression in different cell types at day 6. **e)** *DCN* (magenta) and *RXRA* (yellow), represents the SWAT and adipogenic branch, respectively. FISH analysis partly supported the single cell data showing expression in different cell types at day 4. **f)** *EGR1* (magenta) and *ADIPOQ* (yellow), represents the SWAT and adipogenic branch, respectively. FISH analysis supported the single cell data showing expression in different cell types at day 4. **g)** Positive and negative controls.

Taken together, these data show that the L branch is expressing extracellular matrix and developmental growth factors and is diverging from the adipogenic cells via a competing osteoblast transcription factor network. Our findings were confirmed and complemented by a parallel study from Yang Loureiro et al.^34^. Based on our collective data, we defined these cells as SWAT cells. Hence, the SWAT cells represent a cellular subpopulation that differentiates in a separate direction from the adipogenic cells despite the addition of an adipogenic cocktail. To validate and visualize the separation between the selective transcription factors for the adipogenic and SWAT cells at T5 of human brown adipocytes, we performed a fluorescence in situ hybridization (FISH) analysis using RNAscope probes. We found a clear separation between several transcription factors including the adipogenic transcription factors *SREBF1* and *NR1H3* versus the SWAT *PRRX1*, and the adipogenic transcription factors *PPARG* and *CEBPA* versus the SWAT *SNAI2* (Fig. 2b). We co-stained for additional branching transcription factors, demonstrating a similar cell population separation, except for *JUNB* and *PPARG*, which were expressed in the same cells (Extended Data Fig. 2).

By taking advantage of our pseudotime-ordered dataset and applying a tool called Scellnetor^35^, we identified transcriptional networks involving a subset of the adipogenic cell selective transcription factors (Fig. 2c). Scellnetor is designed to identify gene networks enriched in differentiation trajectories^35^. This network analysis identified additional genes in the adipogenic transcription factor networks including *PPARGC1A*, *PPARGCIB*, *CIDEA* and *CKMT1B*, further emphasizing the adipogenic nature of this branch^36^. We next assessed whether there were any differences in the transcription factor dynamics between BAT-derived and WAT-derived cells. As with the secreted factors, we grouped cells derived from supraclavicular and perirenal brown adipose depots into a ‘BAT’ group and the cells derived from subcutaneous and visceral white adipose depots into a ‘WAT’ group and compared the pseudotime windows at which transcription factors diverged in expression between the adipogenic and SWAT branches (Fig. 2d). For example, we found that *EBF2*, encoding a transcription factor well described for promoting brown adipogenesis^37^ defined the adipogenic branch in BAT-derived cells only, and rather early in pseudotime. However, most differences between BAT-derived and WAT-derived cells became clear later in pseudotime, for example, *RXRG*, which also defined the adipogenic branch in the BAT-derived cells only. RXRG acts as a co-transcription factor with PPARG to promote thermogenic gene transcription^36^ (Fig. 2d).

Among the genes defining the SWAT branch were the above-mentioned *SNAI2* and *JUNB*, driving this branch in both BAT-derived and WAT-derived cells. Interestingly, in late pseudotime, we observed *NFIA*, a SWAT branch-selective gene in brown adipocytes only, previously reported to promote the brown fat differentiation programme^38^ (Fig. 2d). By predefining the cell origin from either BAT or WAT before sorting into pseudotime, we could identify branch-specific transcription factors within these adipose tissue types (Fig. 2d and Supplementary Table 6). Some of these transcription factors, selective for either BAT or WAT derived cells, were reported previously in bulk data^22,36,38^, whereas several are new and might be powerful as directors of the brown and white adipocyte differentiation programmes.

### Defining the adipogenic and SWAT cell signatures

**Fig. 3:**
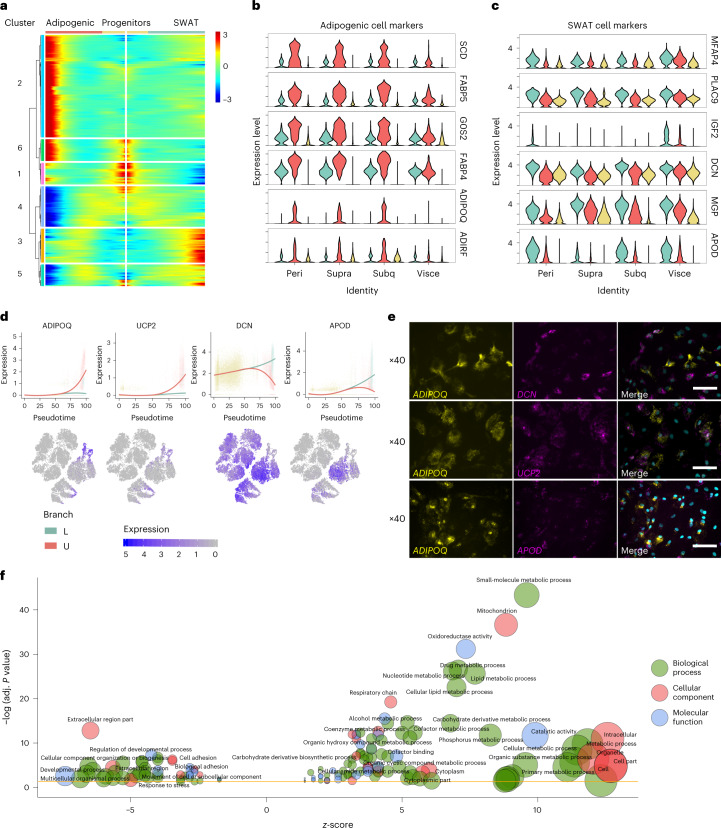
Gene cluster signatures defining adipogenic and SWAT cell branches. **a**, BEAM analysis identified six kinetic clusters of branch-dependent genes. **b**, Violin plots of adipogenic marker genes from cluster 2, showing gene expression in branches across depots. **c**, SWAT cell marker genes from cluster 3, showing gene expression in branches across depots. **d**, Expression dynamics are displayed as a function of pseudotime (stretched, ranging from 0 to 100) of marker genes for the U branch (*ADIPOQ*, *UCP2*) and the L branch (*DCN*, *APOD*). Solid lines show smoothed expression curves for each branch. **e**, FISH staining of human brown adipocytes collected at T5 (halfway through full maturation) using RNAscope probes for branch marker genes. Scale bar, 75 µm. **f**, GO term enrichment analysis visualized using REViGO and the GOplot R package.

We explored the signatures of the adipogenic and the SWAT cells by using branched expression analysis modelling (BEAM)^39^, a bioinformatic approach to identify branch-dependent genes (Supplementary Table 7). We identified six gene clusters of branch-dependent genes with distinct kinetic expression profiles (Fig. 3a). The main cluster upregulated in the adipogenic cells was cluster 2. The top-regulated genes in this cluster comprised genes with well-known roles in adipose tissue functions, including *SCD*, which is important in lipid biosynthesis, the fatty acid carriers *FABP4* and *FABP5* and *ADIPOQ*, encoding adiponectin, a major regulator of lipid metabolism and insulin sensitivity^40^ (Fig. 3b).

The main upregulated cluster in the SWAT cells was cluster 3. The top-regulated genes in this cluster were all locally secreted including the extracellular matrix genes *MGP* and *DCN*, growth factor *IGF2* and *APOD*, encoding apolipoprotein D, which is also present in the extracellular region and is a component of high-density lipoprotein (Fig. 3c). The adipogenic versus SWAT transcription factor profiles were clearly divided between cluster 2 and cluster 3 (Supplementary Table 8). To visualize the regulation of some of the branch-specific genes, we show the feature plots and expression in pseudotime (Fig. 3d). Interestingly, mitochondrial uncoupling protein 2 (*UCP2*) was also upregulated in the adipogenic cells (Fig. 3d). The structure of *UCP2* is similar to that of the brown-fat-specific, thermogenic gene *UCP1*, which was not expressed in the cells at this point of differentiation. To validate and visually examine the separation of the two cell fates within single-cell cultures, we next performed FISH analyses, using RNAscope probes. As predicted, we observed a clear separation between our main markers, *ADIPOQ* and *DCN*, as well as between cells expressing *ADIPOQ* and *APOD*, whereas cells expressing *ADIPOQ* and *UCP2* were overlapping as expected (Fig. 3e).

We performed a Gene Ontology (GO) analysis including all BEAM clusters that defined the adipogenic and the SWAT branches. Multiple genes encoding proteins localizing to the mitochondria and being part of lipid metabolism and the respiratory chain accumulated in the adipogenic cells (Fig. 3f and Supplementary Table 9). On the other hand, the SWAT cells were clearly associated with an accumulation of processes related to extracellular matrix formation, regulation of developmental processes and cellular adhesion (Fig. 3f and Supplementary Table 9).

### The mitochondrial signature of adipogenic cells

**Fig. 4:**
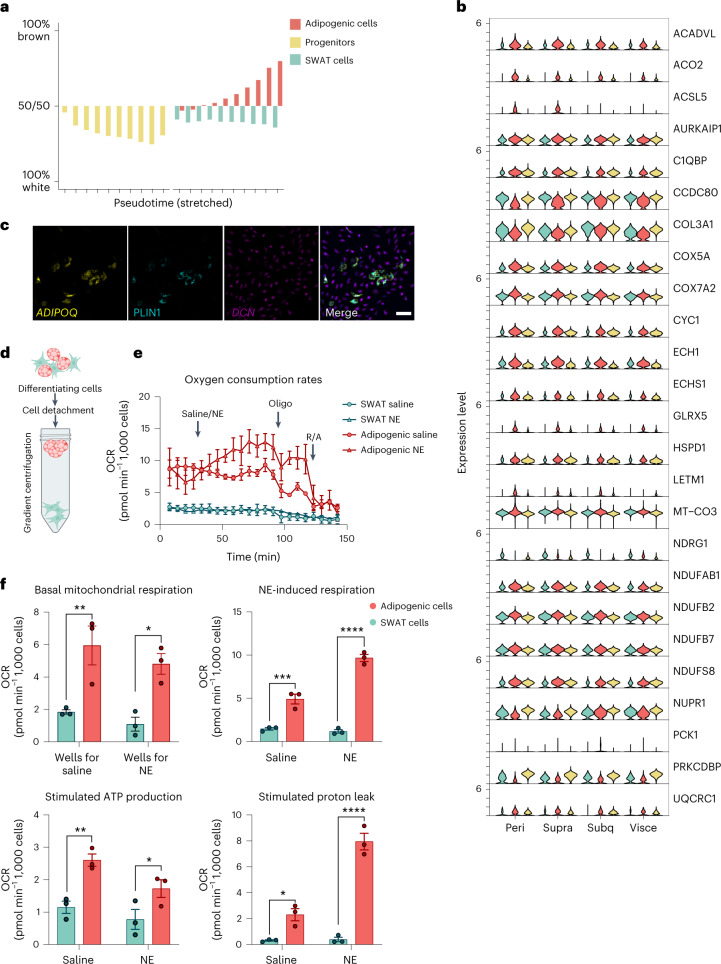
Mitochondrial signature and oxidative capacity of adipogenic compared to SWAT cells. **a**, Predicted brown and white adipocyte content in pseudotime trajectories using BATLAS. Cells are grouped by each developmental branch and pseudotime decile. **b**, BATLAS genes overlapping with this work’s scRNA-seq dataset, expressed in branches across depot origins. **c**, Adipogenic cells contain lipid droplets as indicated by immunofluorescence staining of differentiating adipocytes for perilipin (green) combined with FISH RNAscope for *DCN* (magenta) and *ADIPOQ* (yellow). Nuclei in blue. Scale bar, 100 μm. **d**, Illustration demonstrating density gradient centrifugation to enrich adipogenic and SWAT cells from heterogeneous cultures. Created with BioRender.com. **e**, Seahorse extracellular flux analysis of enriched adipogenic and SWAT cells separated on day 10 of differentiation. Oxygen consumption rates (OCRs) were measured 24 h after plating in DM (5,000 cells per well) and normalized to cell count. *N* = 3 biologically independent cell samples, cultured separately in parallel for 24 h following density gradient centrifugation between SWAT and adipogenic cells. **f**, Calculations of the data visualized in **e**. Two-way analysis of variance (ANOVA) was used to assess differences between cell types and NE treatment in: basal mitochondrial respiration (effect of cell type *P* = 0.0006; effect of NE: *P* = 0.222), NE-stimulated mitochondrial respiration (effect of cell type *P* = 0.0003; effect of NE: *P* < 0.0001), stimulated ATP production (effect of cell type: *P* = 0.0012; effect of NE: *P* = 0.3310) and stimulated proton leak (effect of cell type: *P* < 0.0001; effect of NE: *P* = 0.0001). *N* = 3 biologically independent cell samples, cultured separately in parallel for 24 h following density gradient centrifugation between SWAT and adipogenic cells. Sidak’s post hoc test was used for comparisons between cell types, and significance values are shown in the graphs *P < 0.05, **P < 0.01, ***P < 0.001, ****P < 0.0001. Data are presented as the mean ± s.e.m. NE, noradrenaline; oligo, oligomycin; R, rotenone; A, antimycin; DM, differentiation medium 2.

Given the striking difference in mitochondrial gene expression between adipogenic cells and SWAT cells and the higher expression of mitochondrial genes in BAT compared to WAT, we examined how the branch signatures resonated specifically among marker genes for BAT activity, using the BATLAS^41^ tool. BATLAS is a computational prediction model for identifying brown versus white fat phenotypes in samples of unspecified content. We grouped the cells by each branch, divided them into pseudotime deciles and utilized the BATLAS tool to predict the brown fat signature (Fig. 4a and Supplementary Table 10). We observed that the adipogenic cells obtained an increasingly higher prediction score for a thermogenic phenotype in a pseudotime-dependent manner, while the SWAT cells did not (Fig. 4a).

As shown in ref. ^14^, only hASPCs derived from BAT, but not WAT, develop into thermogenic adipocytes when using our standard differentiation protocol^23^. To address whether the ‘BATLAS signature’ was driven by cells derived from BAT in our dataset, we plotted all genes that were enriched in the BEAM analysis and were overlapping with the BATLAS genes. However, we found a striking consistency in the gene expression patterns between cells from the different depots (Fig. 4b). The only exception was *ACSL5*, encoding long-chain fatty acid CoA ligase 5. This gene was only expressed in the adipogenic cells from BAT but not in any branch in cells derived from WAT. It is important to stress that the BATLAS tool is defined based on thermogenic signatures in tissue samples and mature adipocytes, not on cells in early differentiation. Notably, most of the subset of BATLAS genes overlapping with our dataset were mitochondrial enzymes or subunits in the mitochondrial respiratory chain. Upregulation of mitochondrial metabolism has been found to be crucial for adipogenesis to occur^42^. Importantly, mitochondria binding to lipid droplets provide energy for the lipid droplets to expand^43^. Therefore, given the consistent contribution across all depots, we conclude that the increased expression of the subset of BATLAS genes in our dataset represents a feature of adipogenesis rather than a BAT signature and that this feature is clearly assigned to the adipogenic cells and not present in the SWAT cells.

As adipocytes mature, brown adipocytes maintain a large number of mitochondria, which acquire a thermogenic capacity. To assess the mitochondrial function of adipogenic versus SWAT cells in fully differentiated cells, we therefore aimed to measure the mitochondrial activity in adipogenic cells versus SWAT cells derived from BAT. By co-staining with *ADIPOQ*, *DCN* and perilipin, we observed that adipogenic, but not SWAT cells, were positive for perilipin, confirming that only adipogenic cells contain lipid droplets (Fig. 4c). This allowed us to separate the cell types by taking advantage of their different density. We differentiated hASPCs derived from the supraclavicular BAT depot for 10 d. Cells were detached and the adipogenic cells were separated from the SWAT cells by density gradient centrifugation^34^ (Fig. 4d). The separated cells were plated and, following 24 h in culture, assessed for oxygen consumption using the Seahorse Bioscience technology. We observed a remarkable difference in oxygen consumption between the adipogenic cells and the SWAT cells (Fig. 4e,f). Interestingly, the adipogenic cells not only were more responsive to noradrenaline, but also had a substantially higher respiration at basal level compared to the SWAT cells (Fig. 4g). In conclusion, we assign the high mitochondrial activity needed for adipogenesis to be restricted to the adipogenic cells in a similar fashion across depots. This functional distinction remained in fully differentiated BAT-derived cells.

### SWAT cells are multipotent

**Fig. 5:**
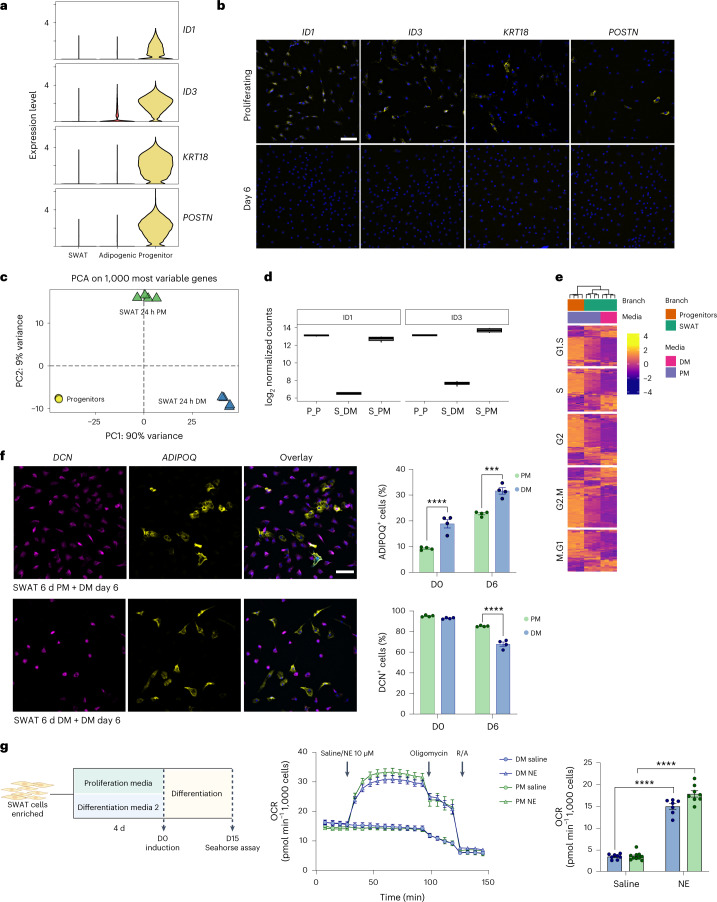
Multipotency of SWAT cells. **a**, Progenitor marker gene expression across branches in scRNA-seq data. **b**, FISH RNAscope staining validating the expression of selected progenitor markers (yellow) in proliferating brown progenitor cells but not in differentiating cells (day 6). Nuclei in blue. Scale bar, 100 µm. **c**, PCA of bulk RNA-seq samples based on the 1,000 most variable genes. **d**, RNA-seq normalized gene counts for selected progenitor markers across conditions. The centre of the box plot is the median of normalized gene expression from *N* = 4 replicates, expressed in log_2_ scale. The lower and upper hinges of the box plot are the first and third quartiles (25th and 75th percentiles), respectively. The whiskers extend from the lower/upper hinges to the smallest/largest values less than 1.5 times the interquartile range (distance between 1st and 3rd quartiles). *N* = 4 biologically independent samples, derived from four separate heterogeneous cell cultures, separated with four individual density gradients and subsequently cultured separately. **e**, Heat map of cell cycle gene expression across conditions, split by cell cycle phase. **f**, Enriched brown SWAT cells on day 12 of differentiation were seeded until they reached sub-confluence in either PM or DM for 6 d and then induced for differentiation. FISH staining confirmed the development of adipogenic (yellow) and SWAT (magenta) cells in both cultures. Nuclei in blue. Scale bar, 100 µm. Two-way ANOVA was used to assess the effects of differentiation in the two groups cultured in different media before differentiation. Top, quantification of *ADIPOQ*-positive cells (effect of differentiation: *P* < 0.0001; effect of cell culture media: *P* < 0.0001). Bottom, quantification of *DCN*-positive cells (effect of differentiation: *P* < 0.0001; effect of cell culture media: *P* = 0.0001). *N* = 4 biologically independent cell samples, cultured separately in parallel for 24 h following density gradient centrifugation between SWAT and adipogenic cells. Sidak’s post hoc test was used for comparisons between cell types, and significance values are shown in the graphs *P < 0.05, **P < 0.01, ***P < 0.001, ****P < 0.0001. Data are presented as the mean ± s.e.m. **g**, Experimental outline to assess the functionality of brown adipogenic cells developed from differentiated SWAT cells seeded in PM or DM (left). Created with BioRender.com. Seahorse extracellular flux analysis (right). Oxygen consumption rates were normalized to cell count. *N* numbers are biologically independent cell samples, cultured and differentiated separately in parallel following density gradient centrifugation between SWAT and adipogenic cells. Left, *N* = 10 for PM-cultured samples. *N* = 8 for DM-cultured samples. Right, two PM-cultured samples and one DM-cultured sample stimulated with NE were excluded due to issues with oligomycin injections, resulting in *N* = 8 for PM and *N* = 7 for DM. Right, calculated stimulated proton leak. Two-way ANOVA analyses was used to assess the effects of NE in the two groups cultured in different cell culture media before differentiation (effect of NE: *P* < 0.0001; effect of cell culture media: *P* = 0.0051). Sidak’s post hoc test was used for comparisons between NE and saline treatment, and significance values are shown in the graphs *P < 0.05, **P < 0.01, ***P < 0.001, ****P < 0.0001. Data are presented as the mean ± s.e.m. *DCN*, decorin; *ADIPOQ*, adiponectin; PM, proliferation medium. Two-way ANOVA with Sidak’s post hoc test *P < 0.05, **P < 0.01, ***P < 0.001, ****P < 0.0001. Data are presented as the mean ± s.e.m.

**Extended Data Fig. 3:**
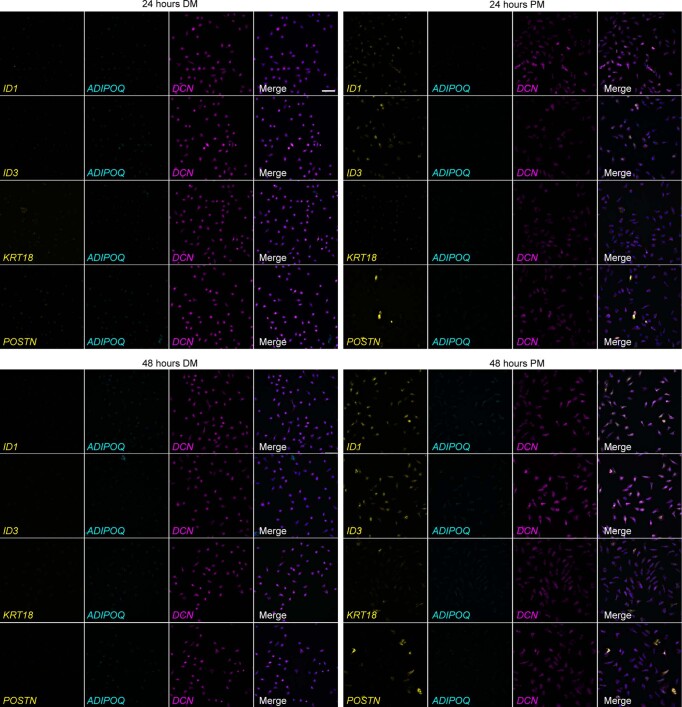
Enriched SWAT cells incubated 24 h and 48 h in proliferation media. SWAT cells were seeded in proliferation media (PM) or Differentiation media 2 (DM) for 24 or 48 hours and co-stained for progenitor markers (*ID1*, *ID3*, *POSTN*, *KRT18*) in yellow, *DCN* (magenta) and *ADIPOQ* (cyan). Nuclei in blue. Scale bar = 100 μM.

We next examined the differences between progenitors and SWAT cells. We first identified selective markers for the progenitors including *ID1*, *ID3*, *KRT18* and *POSTN* (Fig. 5a). These markers were no longer detectable on day 6 of differentiation in any of the cell types (Fig. 5b). We used density centrifugation separation to enrich for SWAT cells on day 12 of differentiation. When plating the SWAT cells in proliferation medium, we observed an increasingly stronger expression of the progenitor markers following 24 h and 48 h incubation (Extended Data Fig. 3), indicating that SWAT cells could revert towards a progenitor-like state. We next examined this at a global level by enriching for the SWAT cells, but this time incubating them either in proliferation medium or in differentiation medium for 24 h, and subsequently performing RNA-seq (Fig. 5c). This experiment demonstrated a clear separation between progenitors and SWAT cells (Fig. 5c). Remarkably, it also showed that switching the cell culture medium back to proliferation medium, partly rewired the cells towards a progenitor-like state, as became evident from the expression of the progenitor markers *ID1* and *ID3* (Fig. 5d). In addition, when specifically monitoring cell cycle genes, the SWAT cells cultured for 24 h in proliferation medium clustered in between the SWAT cells in differentiation medium and the progenitor cells (Fig. 5e).

To examine if SWAT cells, which had reverted to a progenitor-like state, would redifferentiate into adipogenic cells, we divided enriched SWAT cells into two batches, where one batch was plated in proliferation medium and one batch in differentiation medium. The SWAT cells cultured in proliferation medium adopted a progenitor-like state and proliferated until confluence 6 d later when differentiation medium was added (Extended Data Fig. 3). The SWAT cells kept on differentiation medium did not proliferate, but differentiation was initiated at the same time as the corresponding batch in proliferation medium (Extended Data Fig. 3b). Following 6 d of differentiation, *ADIPOQ*-positive cells were present in both SWAT batches (Fig. 5f). The SWAT cells initially maintained in differentiation medium had a higher percentage of *ADIPOQ*-positive adipogenic cells already at induction of differentiation, while after 6 d, both batches had increased percentages of adipogenic cells. The batch in differentiation medium also had a lower percentage of *DCN*-positive cells compared to the batch in proliferation medium.

We next investigated if SWAT cells could differentiate into functional adipocytes. SWAT cells were again enriched without adipogenic cells, and this time, they were incubated for 4 d (until the proliferating batch reached 2 d after confluence), where differentiation medium was added. The cells were differentiated for 15 d, and the oxygen consumption rate was measured using the Seahorse Bioscience technology, clearly demonstrating noradrenaline responsiveness and increased stimulated proton leak in both batches (Fig. 5g). In conclusion, these findings suggest that SWAT cells are multipotent and responsive to their microenvironment.

### A common progenitor of adipogenic and SWAT cells

**Fig. 6:**
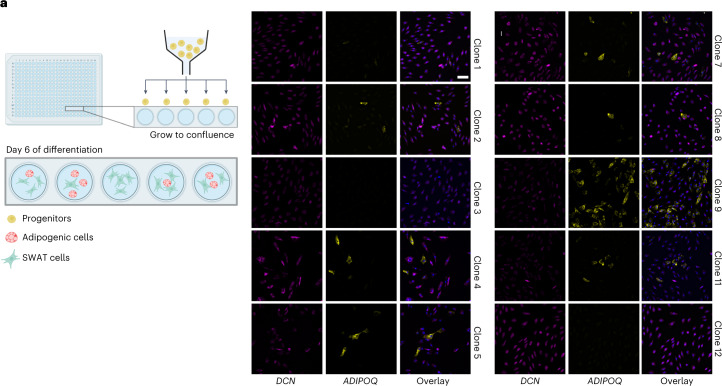
FACS-based cloning of human adipose stem and progenitor cells. **a**, Graphic illustration of single-cell-sorted progenitors with subsequent differentiation of the clonal populations. Created with BioRender.com. Clonal populations developed into adipogenic (yellow) and SWAT (magenta) cells as visualized with FISH RNAscope staining on day 6 of differentiation. Nuclei in blue. Scale bar, 100 µm.

Our computational data suggested that SWAT cells and adipogenic cells differentiated from a common progenitor cell. To validate this finding, we set up a clonal expansion experiment using FACS sorting. Single progenitor cells were sorted with a FACS Melody Cell Sorter into 96-well plates. After 2 weeks in culture, clonal populations that reached 70–90% confluency were selected and further expanded until differentiation was induced according to our standard protocol. The cells were fixed on day six of differentiation and stained for adipogenic (*ADIPOQ*) and SWAT (*DCN*) markers. Ten of the expanded clonal populations are shown (Fig. 6a), demonstrating that most clones developed into both adipogenic and SWAT cells, whereas a minority of clones only demonstrated SWAT cells. The ratio beween adipogenic and SWAT cells differed across clones, suggesting some stochasticity in the determination of branching. In conclusion, these data confirm our computational prediction that SWAT cells and adipogenic cells arise from a common progenitor.

### In vivo relevance of SWAT cells, adipogenic cells and progenitor cells

**Fig. 7:**
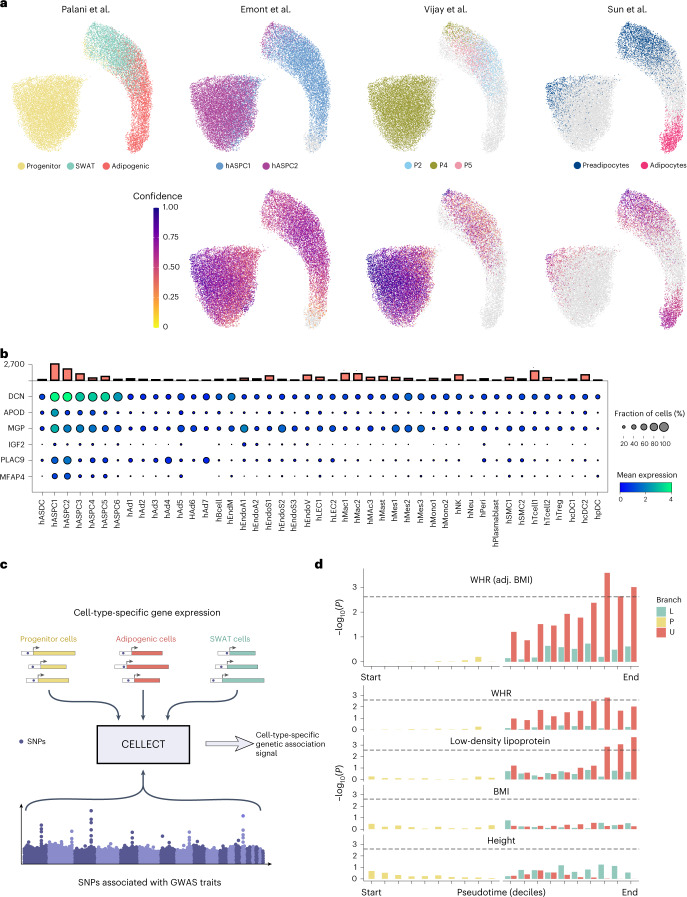
Label transfer to human adipose tissue and genome-wide association study traits. **a**, Uniform manifold approximation and projection dimensionality reduction scatterplots of cells from Palani et al., scRNA-seq (this work) mapped with labels transferred from reference datasets, as annotated in the figure, using Scanorama. Confidence scores are the probability of label assignment by *k*-nearest neighbours (kNN) classifier. **b**, Dot plot displaying the expression of top markers for SWAT cells in the ASPC subpopulations as defined^9^. Bar plot on top indicates the number of cells associated with each cell-type annotation. **c**, CELLECT tool method. **d**, CELLECT analysis of progenitor, U (adipogenic) and L (SWAT) branch cells, binned by pseudotime deciles. Adipogenic cells with the highest pseudotime values showed significant association with WHR (adjusted to BMI) and low-density lipoprotein. Other traits did not reveal any association.

**Extended Data Fig. 4:**
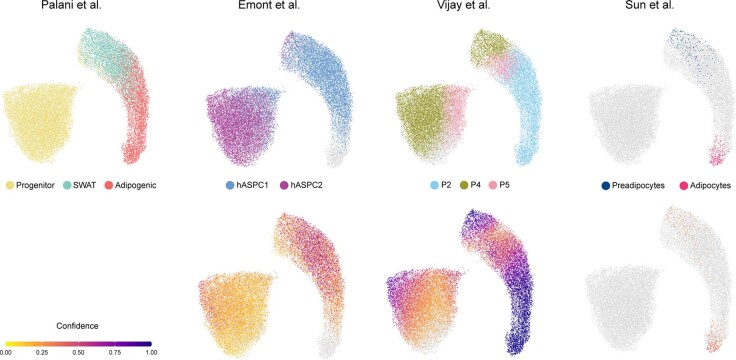
Label transfer using scNym. UMAP dimensionality reduction scatterplots of cells from Palani et al., scRNA-seq (this work) mapped with labels transferred from reference datasets, as annotated in the figure, using scNym.

To address how our dataset compared to recent human studies, we used two different machine learning-based algorithms for projecting published datasets^9,44,45^ onto our data space. The Scanorama algorithm was recently highlighted as a suitable method for comparing datasets of variable origin^46^ (Fig. 7a), whereas scNym produced similar results (Extended Data Fig. 4). In the human subcutaneous and visceral adipose tissue single-nucleus RNA-seq (snRNA-seq) dataset from Emont et al.^9^, two separate populations mapped to our data. The population classified as hASPC2 mapped to our progenitor population, whereas the population classified as hASPC1 mapped to both SWAT and adipogenic cell branches. Projecting a dataset comprising scRNA-seq of the stromal vascular fraction of human adipose tissue from Vijay et al.^44^, identified a match between the ‘P4’ population and our progenitor population, whereas the SWAT cells were mostly matched to ‘P5’ but intermixed with ‘P4’ and the adipogenic cells matching mostly to ‘P2’. Finally, a human BAT snRNA-seq dataset from Sun et al.^45^ projected ‘preadipocytes’ to the SWAT population and ‘adipocytes’ to the adipogenic population (Fig. 7a and Extended Data Fig. 4). In conclusion, all three cell types could be found computationally with populations described in single-cell or snRNA-seq datasets of human WAT and BAT.

**Extended Data Fig. 5:**
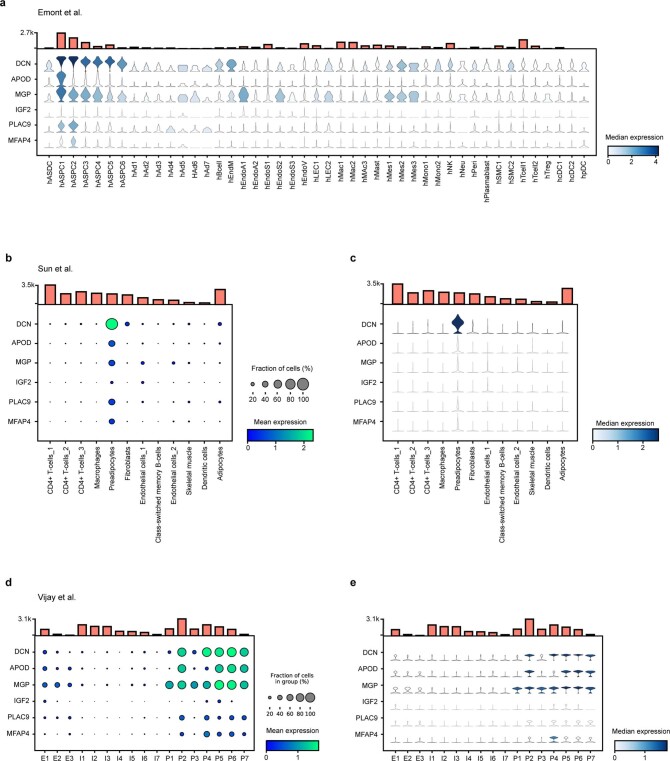
SWAT markers in previously defined APSC subpopulations. **a)** Violin plot displaying the expression of top markers for SWAT cells in the ASPC subpopulations as defined (10). Bar plots on top indicates the number of cells associated with each cell type annotation. **b)** Dot plot and **c**) Violin plot displaying the expression of top markers for SWAT cells in a BAT single nuclei data set as defined (46). Bar plots on top indicates the number of cells associated with each cell type annotation. **d)** Dot plot and **e)** Violin plot displaying the expression of top markers for SWAT cells in a stromal vascular fraction data set as defined (45). Bar plots on top indicates the number of cells associated with each cell type annotation.

The multipotency of SWAT cells and the overlaps between annotated populations raised the idea of potential subtypes annotated in the in vivo datasets. To investigate this, we mapped the top SWAT markers from Fig. 3a within the three datasets (Fig. 7b and Extended Data Fig. 5a–e). This comparison was consistent with the data label transfer results, but also showed that additional hASPC populations^9^ had some SWAT cell marker gene expression, potentially representing SWAT subtypes or states (Fig. 7b and Extended Data Fig. 5a). In BAT^45^, SWAT cell top markers mapped consistently to the preadipocytes (Fig. 7c,d). Interestingly, when comparing to the stromal vascular fraction datasets^44^, P2 and P4–P6 resembled SWAT cells (Extended Data Fig. 5d,e). This indicates that SWAT cells might appear at different maturity states in vivo as P2 was described to represent a more mature cell type compared to P4–P6 (ref. ^44^).

To investigate the relevance of our cell types in human health and disease, we next used the computational tool CELLECT, to prioritize our pseudo-temporal ordered data for relevant human traits. Briefly, CELLECT quantifies the association between the expression patterns of cell types and the genetic components of human complex traits identified by genome-wide association studies (GWAS). CELLECT tests for enrichment of genetic association signal for single nucleotide polymorphisms (SNPs) proximal to genes specifically expressed in each cell type. In a previous study of human adipose tissue, CELLECT identified an association between adipocytes and traits for waist-to-hip ratio (WHR)^9^. With our dataset, we integrated a temporal aspect during the early differentiation of adipocytes into the analysis. To do this, we stratified cells by pseudotime decile for each developmental branch, generating 30 strata of cells. Cells from each stratum were then used as input to CELLECT to estimate expression specificity of genes in any of these groups. We performed CELLECT analysis for 39 GWAS traits (Supplementary Table 11). We identified significant enrichment of genetic association signal for cell populations in the adipogenic branch for fat distribution (as assessed by WHR and WHR adjusted for BMI), and lipid levels (as assessed by low-density lipid levels; Fig. 7b and Supplementary Table 12). The progenitors and SWAT branches displayed little or no enrichment. As a control of the model, we assessed the genetic enrichment for height and BMI GWAS and found no enrichment, consistent with the expectation that adipogenic development does not affect human height. Neither did we find enrichment for any of the remaining GWAS traits analysed.

These results indicate that the level of onset of adipogenic genes would determine the balance between SWAT cells and adipogenic cells across adipose depots, and this branching might thus be relevant for determining fat distribution in humans.

## Discussion

We investigated the first phase of adipogenesis at single-cell resolution across cells derived from four different human BAT and WAT depots. We demonstrate that two cell types arise from a common progenitor cell at the onset of differentiation: a classical adipogenic cell type, and an alternative cell type that we define in this paper, with a signature of extracellular matrix-secreting and developmental genes. Our findings are consistent with the data of Yang Loureiro et al.^34^, who observe the same early split during adipogenesis in progenitor cells, and demonstrate that Wnt signalling defends a multipotent state in the alternative cell type. Based on our collective data, we named these cells SWAT cells.

A major finding in the current study is that SWAT cells and adipogenic cells arise from a common progenitor. The branching between the two cell types is induced after adipogenic development, where the SWAT cells avoid adipogenic differentiation by upregulating an osteoblast transcription network^4^, instead of inducing the competing adipogenic transcription programme. Yang Loureiro et al. demonstrate that the SWAT cells are defined by a rapid upregulation of Wnt signalling following the onset of differentiation. SWAT cell identity is dependent on the microenvironment and appears to be maintained by intercellular cross-talk. To this end, Yang Loureiro et al. identified pairs of extracellular matrix components from the SWAT cells and their corresponding receptors on the adipogenic cells, whereas we observed that SWAT cells effectively differentiate into adipocytes when the existing adipocytes were stripped from the cultures. This suggests that an intercellular cross-talk might keep the balance between the two cell types.

Our in vitro approach allowed us to study early differentiation and detect two cell trajectories that would have been difficult to observe in tissue samples. However, a limitation of our study was the possibility that this branching was a cell culture artifact. Yang Loureiro and co-authors addressed this by transplanting human progenitor cells into immune-compromised mice, observing that both SWAT and adipogenic cell signatures develop from these transplants. This experiment demonstrates that branching occurs in vivo. In addition, we observed strong indications that SWAT cells also exist in vivo in humans by comparing with published datasets. SWAT cells resemble cell populations identified in several single-cell studies of adipose tissue^9,11,44,45,47^.

Interestingly, we found that the SWAT cells mapped to the hASPC2 population annotated in Emont et al.^9^. This is intriguing as hASPC2s are similar to a population of multipotent progenitors traced to the interstitial reticulum in murine adipose tissue^48^. In support of a multipotent SWAT cell population present in vivo, we found that several ASPCs subtypes, annotated in vivo, expressed SWAT markers. This included a subtype localizing near macrophages and vascular and fibrotic structures^11^. Our findings underscore the in vivo presence of SWAT cells and raise the idea that distinct subtypes or states of SWAT cells are detectable in vivo.

Could the balance between SWAT cells and adipogenic cells have consequences for adipose tissue state in metabolic health and disease? Interestingly, it was shown previously that stimulating beige fat adipogenesis in mice was tipping the balance from a fibrogenic to an adipogenic phenotype^49,50^. Given that beige fat adipogenesis is highly dependent on the adipogenic PPARG-driven transcription factor programme, it is possible that these interventions in fact affected the branching between SWAT and adipogenic cells. SWAT cells express developmental and extracellular matrix genes, many of which are upregulated during obesity-induced adipose tissue fibrosis. Thus, dysregulation of the branching between SWAT and adipogenic cells is a potential source of adipose tissue dysfunction and subsequent development of cardiometabolic disease.

In conclusion, we here present a differentiation map for adipogenic and SWAT cells, arising from a common progenitor across cells derived from four human BAT and WAT depots. Our findings suggest a competing balance between these two cell fates with complementing roles in the adipose tissue architecture and function. From a larger perspective, our study provides a new insight into the connection between cardiometabolic health and adipogenic differentiation across depots.

## Methods

### Human samples

Human adipogenic progenitor cells were isolated from the stromal vascular fraction of adipose tissue samples on the day they were obtained (surgery or biopsy) from four regions: (1) visceral adipose tissue (obtained during gallbladder surgery); (2) perirenal adipose tissue (obtained during nephrectomy surgery); (3) abdominal subcutaneous adipose tissue (obtained with the Bergström needle biopsy method); and (4) supraclavicular adipose tissue (obtained during surgery in patients with suspected cancer of the neck). Isolated cells were expanded and frozen in liquid nitrogen in a proliferative state until the onset of the study. Data from the cohorts have previously been published^14–16^. All participants provided written consent and the studies were performed in accordance with the Declaration of Helsinki. The cell studies were approved by the Danish Data Protection Agency, Denmark (journal no. RH-2017-69, I-suite no. 05329).

### Cell culturing

Biopsy samples were collected in DMEM/F12 (11039047, Gibco) with 1% penicillin–streptomycin (15140122, Gibco) and tubes were kept on ice during transport. A detailed protocol for isolation and culturing of human adipocyte progenitors has been previously contributed^23^. Briefly, biopsy samples were digested with 10 mg collagenase II (C6885-1G, Sigma) and 100 mg BSA (A8806-5G, Sigma) in 10 ml DMEM/F12 for 20 min at 37 °C while gently shaken. Following digestion, the suspension was filtered, and cells were washed with DMEM/F12, resuspended in DMEM/F12, 1% penicillin–streptomycin, 10% FBS (10270-106, Gibco) and seeded in a 25-cm^2^ culture flask. Medium was changed the day following isolation and then every second day until cells were 80% confluent; at this point, cultures were split into a 10-cm dish (passage 0). Cells were expanded by splitting at a 1:3 ratio. For the single-cell experiment, cells were seeded in six-well plates in proliferation medium consisting of DMEM/F12, 10% FBS, 1% penicillin–streptomycin and 1 nM fibroblast growth factor-acidic (FGF-1; 11343557, ImmunoTools). Cells were grown at 37 °C in an atmosphere of 5% CO_2_ and the medium was changed every second day. Adipocyte differentiation was induced 2 d after adipocyte progenitor cultures were 100% confluent, by removal of FGF-1 and FBS from the medium and addition of a differentiation cocktail. This cocktail consisted of DMEM/F12 containing 1% penicillin–streptomycin, 0.1 μM dexamethasone (D490, Sigma), 100 nM Actrapid insulin (A10AB01, Novo Nordisk), 200 nM rosiglitazone (R2408, Sigma), 540 μM isobutylmethylxanthine (I5879, Sigma), 2 nM T3 (T5516, Sigma)) and 10 μg ml^−1^ transferrin (T8158, Sigma). After 3 d of differentiation, isobutylmethylxanthine was removed from the cell culture medium and cells were differentiated for an additional 3 d with the remaining differentiation compounds. For cells differentiated until a fully mature state, differentiation medium 2 (differentiation cocktail without rosiglitazone and isobutylmethylxanthine) was applied from day 6 onwards and changed every third day. For the 10x single-cell sorting, cells were loosened by adding 2 ml of TrypLE Express Enzyme (12605010, Gibco) and placed in incubator for 3 min. Detachment of cells was confirmed by microscopy and 3 ml of proliferation medium was added to the cells to inactivate trypsin. Next, 190 μl of cell suspension was then transferred to a microcentrifuge tube and mixed with 10 µl of Solution 13 AO-DAPI (910-3013, Chemometec), and then counted on a nucleocounter NC-3000 (Chemometec). Cells were counted as described above, and 8,000 cells per donor were pooled in a microcentrifuge tube. The pool of cells was centrifuged for 7 min at 700 *g* and resuspended in 80 µl PBS with fatty acid-free BSA.

### Cell and molecular biology methods

#### RNA FISH

In vitro differentiated human adipocytes derived from either subcutaneous or supraclavicular deep neck adipose depots were fixed on days 4 and 6 of differentiation with 10% neutral buffered formalin (HT501128-4L, Sigma) for 30 min, dehydrated and stored in 100% ethanol until the staining procedure. In situ hybridization was performed using RNAscope Multiplex Fluorescent Detection Kit v2 and RNAscope 4-Plex Ancillary Kit for Multiplex Fluorescent Kit v2 (323110 and 233120, ACDbio). RNAscope manual assay probes were designed and produced by Advanced Cell Diagnostics. Nuclei were stained with NucBlue ReadyProbes (R37605, Invitrogen). RNA targets were visualized using an EVOS imaging system if not stated differently (Invitrogen). The RNA targets were hybridized with RNAscope probes (Supplementary Table 13) and then labelled with Opal 520 (FP1487001KT), Opal 570 (FP1488001KT), Opal 620 (FP1495001KT) and Opal 690 (FP1497001KT; all Akoya Biosciences). The fluorescence signals were detected with RFP, Cy5 and Texas Red light cubes.

### Identification and validation of progenitor markers

ASPCs from the supraclavicular BAT of the same donor were fixed in the proliferating phase or on day 6 of differentiation as described in ‘RNA FISH’ and stained for *ID1*, *ID3*, *POSTN* and *KRT18* RNA expression. Images were acquired with the Leica Thunder at a magnification of ×20.

### Immunofluorescence staining for perilipin staining

Differentiating ASPCs (day 6) from supraclavicular BAT of one donor were fixed in a standard 96-well plate as described in RNA FISH. After performing RNA FISH staining for *DCN* and *ADIPOQ*, the cells were permeabilized with 0.1% Triton X-100 (1003407653, Sigma) in PBS for 10 min at room temperature (RT), blocked with 10% goat serum (G9023, Sigma) in PBS for 60 min at RT and incubated overnight at 4 °C with the primary anti-perilipin antibody (9349S, Cell Signaling) in blocking solution (1:200 dilution). After 3× thorough PBS washes, Alexa Fluor 488-conjugated secondary antibody (A21206, Invitrogen) was diluted at a ratio of 1:750 in PBS and added for 1 h at RT followed by 2× PBS washes. Nuclei were stained with Hoechst 33342 (8 µM; ab228551, Abcam) for 10 min at RT and washed 3× with PBS, whereas the last wash was left on the cells. Images were acquired with the Leica Thunder microscope at a magnification of ×20.

### Percoll density gradient separation

Differentiating adipocytes were enriched for SWAT or adipogenic cells as described in ref. ^34^ and differentiation was extended to 10–12 d to increase the lipid content of the adipogenic cells. In short, a Percoll (4937, Sigma) density gradient was prepared in a 15-ml centrifuge tube by diluting Percoll with differentiation medium 2 to final densities of 1.03, 1.02 and 1.01 g ml^−1^. Differentiating ASPCs were detached with TrypLE, centrifuged for 5 min at 300*g* and resuspended in 500 µl of 1.01 g ml^−1^ Percoll solution. This cell suspension was carefully layered on top of the gradient and centrifuged for 30 min at RT at 1,000*g*. The adipogenic cells were enriched in the floating low-density fraction, while the SWAT cells accumulated in the high-density fraction as pellet. The low-density fractions were carefully removed with a pipette and transferred to a new centrifuge tube, and the high-density pellet was resuspended in differentiation medium 2. Cells were counted with the NucleoCounter NC-3000 (Chemometec) and diluted to the required seeding densities. For experiments where enriched SWAT cells were seeded in either differentiation medium 2 or proliferation medium, the resuspended SWAT fraction was separated into two tubes, centrifuged for 5 min at 1,000*g* and the pellets dissolved in the corresponding medium.

### Differentiation potential of enriched SWAT cells

Enriched SWAT cells were seeded until they reached sub-confluency (10,000 cells per well) in either proliferation or differentiation medium 2 into 96-well plates. To assess the re-appearance of progenitor markers in SWAT cells cultured in proliferation medium versus differentiation medium 2, cells were fixed 24 or 48 h after seeding followed by RNA FISH staining for *ID1*, *ID3*, *POSTN* and *KRT18*. Images were acquired with the Leica Thunder microscope at a magnification of ×20. To investigate the differentiation potential of enriched SWAT cells seeded in proliferation medium or differentiation medium 2, the cells were grown to confluence and subjected to a second round of differentiation (‘Cell culturing’). Cells were fixed on day 0 and day 6 of differentiation and stained for *ADIPOQ* and *DCN* with RNA FISH. Representative images were acquired with the Leica Thunder microscope at ×20 magnification and the formation of *DCN*-positive and *ADIPOQ*-positive cells was quantified with the ImageXpress Pico microscope (Molecular Devices). The thermogenic function of the SWAT cell-derived adipocytes was measured on day 15 of differentiation by measuring noradrenaline-induced respiration using the Seahorse extracellular flux system (Agilent; ‘Extracellular flux analysis with Seahorse and nuclei count’).

### Quantification of *DCN*-positive and *ADIPOQ* -positive cells

The ImageXpress Pico Automated Cell Imaging System from Molecular Devices was used to image and quantify the development of adipogenic cells (*ADIPOQ* positive) and SWAT (*DCN* positive) cells. Cells were fixed on day 6 of differentiation and stained for *DCN* (Cy5) and *ADIPOQ* (Texas Red) expression as described in ‘RNA FISH’. Nuclei were stained with DAPI. Images were acquired with a ×10 objective and 50% of the well area was covered. The CellReporterXpress Analysis Software was used to detect *ADIPOQ*-positive and *DCN*-positive cells based on their respective fluorescence signal with the pre-configured pipeline ‘two-channel assay for scoring cells based on nuclear stain and a marker’. Results are expressed as the percentage of nuclei positive for Cy5 or Texas Red from all counted nuclei. The channels were analysed in two separate analyses and the entire acquisition area was analysed.

### Seahorse experiments with enriched adipogenic and SWAT cells

Mature adipocytes differentiated from the supraclavicular BAT of one donor underwent Percoll density gradient centrifugation on day 10 of differentiation and were seeded on a seahorse 96-well plate (5,000 cells per well). For adipogenic cells, the wells were pre-coated with rat collagen I (3447-020-01, R&D systems) for 1 h at 37 °C in a cell culture incubator to support adherence. Seahorse extracellular flux measurements were performed 24 h after seeding as described below.

### Extracellular flux analysis with Seahorse and nuclei count

Before the extracellular flux measurements, cells were incubated in phenol-free Seahorse XF base media (102353-100, Agilent Technologies) supplemented with 25 mM glucose, 1 mM sodium pyruvate (11360070, Gibco) and 2 mM Glutamax (35050061, Gibco) for 1 h at 37 °C in a non-CO_2_ incubator. After baseline measurements, noradrenaline (10 µM, 745661, Amgros I/S) or PBS (10010023, Gibco) was injected, followed by the injection of 2 µM oligomycin, 0.75 µM FCCP and 0.75 µM rotenone/antimycin from the Mito StressTest kit (103015-100, Agilent Technologies). Seahorse experiments were performed with a Seahorse XFe96 analyzer coupled to a BioTek Cytation 1 to normalize oxygen consumption rates to cell number. To stain the nuclei, Hoechst 33342 (8 µM; ab228551, Abcam) was added after the last oxygen consumption measurement and the plate was incubated for 20 min at RT in the dark.

To estimate the cell count from enriched SWAT and adipogenic cells, the acquired images (BioTek Cytation) were analysed with a programme created in Python 3.10.6 that was able to clean the images for background noise and count the dots representing the individual cells. The main Python site packages used for the programme were opencv-python, scikit-image, Numpy and Matplotlib (see GitHub for references; https://github.com/). The overall steps of the programme include: image reading and conversion to greyscale and Numpy arrays, calculating multi-Otsu thresholds for removal of background noise, detection of all connected components, computation of the maximum size for dots using the kneebow site package, removal of noise based on the hardcoded minimum dot size and the kneebow-calculated maximum size, but with a hardcoded maximum size. In the final step, the cells were counted.

### Bulk RNA-seq of enriched SWAT cells versus progenitor cells

ASPCs from one donor were differentiated until day 12 and enriched for SWAT cells. Each technical replicate represents one density gradient centrifugation, whereas approximately 400,000 cells from the enriched pellet were cultured in proliferation medium or differentiation medium 2 for 24 h and then collected in TRIzol (15596026, Invitrogen). Proliferating ASPCs from the same donor and passage were grown on 10-cm dishes and harvested in TRIzol after 3 d in culture. For RNA isolation, cells were lysed in TRIzol reagent, and a 1/5 volume of chloroform was added for phase separation. Samples were centrifuged at 4 °C at 12,000*g* for 15 min and the aqueous phase carefully transferred to new tubes. Samples were mixed with 70% ethanol at a 1:1 ratio and RNA subsequently isolated with the RNeasy Micro kit (74004, Qiagen) using the Qiagen protocol ‘Clean up after lysis and homogenization with QIAzol lysis reagent’. For bulk RNA-seq, libraries were prepared from 250 ng total RNA with the Universal Plus Total mRNA-Seq library preparation kit with NuQuant (0520-A01, Tecan) and samples were sequenced as paired-end reads on an Illumina NovaSeq 6000 instrument.

### Clonal cell populations

Proliferating ASPCs from the supraclavicular BAT of one donor were detached with TrypLE and centrifuged for 5 min at 1,250 r.p.m. to remove TrypLE. The cell pellet was resuspended in 4 ml of FACS buffer (1 mM EDTA (15575-038 Invitrogen), 2% FBS, 15 mM HEPES (15630-080 Gibco), 0.1 mg ml^−1^ Primocin (InvivoGen)) and filtered through a 40-µm cell strainer to remove potential cell aggregates. The filter was washed once with 4 ml of FACS buffer and cells counted with a NucleoCounter NC-3000 (Chemometec). The cell suspension was centrifuged for 5 min at 300*g* to collect the cells, which were finally concentrated to 1 × 10^6^ cells per ml in FACS buffer. Cells were stained with 2 µg ml^−1^ 7-ADD (59925, BD Pharming) to discriminate between live/dead cells and kept on ice until sorting. Single cells were sorted with a FACS Melody Cell Sorter (BD Biosciences) into Matrigel-coated 96-well plates to support cell attachment. For Matrigel coating, Matrigel (354230, Corning) was diluted to 60 µg ml^−1^ in serum-free medium, distributed into 96-well plates and incubated for 1 h at 37 °C in a cell culture incubator. Unbound Matrigel was removed, plates washed 1× with serum-free media and stored in the incubator until usage. To culture the sorted cells, fresh proliferation media (10% FBS, 0.1 mg ml^−1^ Primocin, FGF-1) was mixed with conditioned medium at a 1:1 ratio. The conditioned medium was harvested and sterile filtered every third day from proliferating APSCs from the same donor grown on 15-cm dishes. After sorting, cells were left undisturbed for 5 d and medium was subsequently changed every third day. After 2 weeks in culture, clonal populations that reached 70–90% confluency were detached with TrypLE and split with a 1:2 ratio on new 96-well plates without coating for further expansion. Differentiation was induced on 2 d post-confluent clonal populations and cells fixed on day 6 of differentiation for RNA FISH staining to investigate the formation of SWAT and adipogenic cells. Microscope images were acquired with the Leica Thunder at ×20 magnification.

### Analysis software and statistics for cell experiments

Data are represented as means ± s.e.m. Statistical analysis was performed with GraphPad Prism version 9.30. One-way or two-way ANOVA with appropriate multiple-comparison adjustments were performed as indicated in the figure legends. *N* values are stated in the figure legends.

### Bioinformatics methods

#### Single-cell library preparation and sequencing

Single-cell cDNA libraries were generated using the Chromium Single Cell platform and 3′ v2 Reagent Kit according to manufacturer’s protocol (10x Genomics). Single-cell libraries were sequenced on a NextSeq 500 platform to obtain 100-bp and 32-bp paired-end reads using the following read length: read 1, 26 cycles; read 2, 98 cycles; and i7 index, 8 cycles. Cell Ranger^51^ (version 2.0.1) was used with default parameters to demultiplex and align reads to the hg19 reference genome, filter cell and unique molecular identifier (UMI) barcodes and generate gene count matrices.

### Genotyping

Samples were genotyped with Infinium Global Screening Array-24 v1.0 (Illumina). Genotypes for each individual were called using Illumina GenomeStudio (v2.0) with h19 as the reference genome. We exported the genotype calls using the PLINK export plug-in (PLINK Input Report Plug-in v2.1.4) and used the software HRC-1000G-check-bim (v4.2.9) to perform quality control on the 618,540 genotyped SNPs before imputation. The programme matches strand, SNP ID names, positions, alleles, ref/alt assignment to 1000 Genome Project reference data. After quality control, 267,821 SNPs were retained. These SNPs (excluding the X chromosome) were imputed using Minimac3 via the Michigan Imputation Server with default settings (EUR 1000 Genomes phase 3 v5 as the reference panel and phasing using Eagle v2.3). Of the 47,109,485 imputed SNPs, we retained 3,031,027 SNPs with high imputation quality (*R*^2^ > 0.8) and minor allele frequency > 0.2.

### Genotyping and Demuxlet sample identity deconvolution

The Demuxlet algorithm^52^ allows for genetic deconvolution of sample identity and doublet detection in single-cell libraries with samples pooled across individuals. We used Demuxlet (version 1.0, downloaded 25 July 2018) and genotypes were obtained as described above to deconvolute sample identity in our cell libraries. As recommended by the authors of Demuxlet, we filtered out SNPs in non-exonic regions (defined by GENCODE release 19), retaining 93,898 SNPs, before running Demuxlet. The average number of SNPs per cell reported by Demuxlet was 127 (counting cells in the Cell Ranger filtered matrices). We discarded 3,444 cells (12.8% of total) identified by Demuxlet as doublets (Supplementary Table 14).

### Proliferating progenitor analysis

We used the R package Seurat^53^ for preprocessing the data, quality control, regression of cell cycle effects, sample alignment and differential expression analyses. We performed quality control on our data to filter out low-quality cells and genes, and we preprocessed the data to the format required for further analysis. As all proliferating adipocyte progenitors could be expected to have similar mitochondrial content, we filtered out cells where the mitochondrial gene expression was higher than 8%, as deviating high mitochondrial gene expression indicates stressed cells. Cells with less than 200 genes or more than 9,000 genes were also removed, as well as cells with more than 120,000 UMIs to remove possible doublets. The filtered data were log normalized and scaled, and the number of UMIs and percentage of mitochondrial genes were subsequently regressed on the data. PCA was performed on the data and the first 15 principal components (PCs) were used for clustering and *t*-SNE visualization. Each cell was then scored for cell cycle phase.

### Differentiating adipocyte analysis

We used the R package Seurat^53^ (version 2.3.4) for preprocessing the data, quality control and differential expression analyses. As input to Seurat, we used the digital gene expression matrix output from the 10x Genomics analysis pipeline Cell Ranger. Cells with less than 200 genes and genes expressed in less than three cells were filtered out. The percentage of mitochondrial gene expression was calculated for each cell. However, as mitochondrial gene expression increases during adipogenesis, we did not exclude cells with high mitochondrial expression in the developing adipocyte progenitor dataset. We performed PCA to compute PCs needed for clustering and data visualization. PCs were computed on the highly variable genes identified on log normalized and scaled data. Clusters were identified using a shared nearest-neighbour modularity optimization-based clustering algorithm, which uses the number of significant PCs as input. The data were visualized using Seurat’s implementation of *t*-SNE^54^ .

### Monocle

We used the R tool Monocle^55^ (version 2.8.0) to construct the cell developmental trajectory of the preprocessed Seurat object. Feature selection for trajectory construction was performed as follows: First, the dataset was split into two subsets, one containing all cells from T1, T2 and T3 and one containing all cells from T4 and T5. Both subsets were then clustered using Seurat’s default clustering algorithm with a resolution of 1.5. Differential expression tests were performed for every cluster against the rest of the cells in the subset (using the negative binomial test, filtering on absolute log fold change > 0.25). The union of the resulting gene list (2,464 genes) was used as the input feature list for building the Monocle trajectory (DDRTree algorithm, max_components = 2). Monocle orders cells by pseudotime along the trajectories. (Pseudotime is an abstract unit of progress: it is the distance between a cell and the start of the trajectory, measured along the shortest path. The trajectories total length is defined in terms of the total amount of transcriptional change that a cell undergoes as it moves from the starting state to the end state.) We used Monocle’s BEAM^39^ to identify branch-dependent genes. The genes in the resulting list were filtered on *q* value < 0.05 (8,647 genes remaining) and subsequently filtered on absolute average log fold change > 0.3 between the U branch and L branch (413 genes remaining). To subset transcription factors in the BEAM analysis, we retrieved the gene type, GO term name and GO term definition for every gene in our dataset using Ensembl Biomart (version 96). From this set of annotated genes, we created two gene sets: a transcription factor gene set by selecting genes annotated with the ‘transcription factor’ GO term, and a non-coding gene set by filtering out genes annotated with the gene type ‘protein_coding’.

### Velocyto

We ran the Velocyto^18^ command line tool for every sample with a genome annotation file and expressed an annotation file of repeats (reference genome hg19). We used the Python library for further downstream analysis. First, the loom files of every sample were aggregated into one. Cells that were not present in the final Seurat analysis were discarded, and the metadata from the Seurat analysis were added to the remaining cells. We further discarded cells with extremely low unspliced detection, keeping 23,309 cells for the final analysis. Genes were filtered by ranking the spliced genes based on a coefficient of variation versus mean fit, using the top 3,000 to perform a PCA. Both the spliced and the unspliced gene expression matrices were subsequently normalized by size. Using the first 15 PCs, the data were smoothed using kNN (using the default value of *k*; 0.025 × *n*Cells). The standard implementation of Velocyto was used with default parameters for fitting gene models, predicting velocity, extrapolating and plotting.

### Gene-set enrichment analyses

Gene-set enrichment analyses were performed using the R package gProfileR^56^. GProfileR takes as input a list with gene symbols and returns a table with terms associated with those genes. We filtered the output to only contain GO terms. To generate the figure with GO terms, we performed gene-set enrichment analyses on every cluster of branch-dependent genes identified using BEAM (6 clusters, 413 genes in total, absolute log fold change > 0.3 between U branch and L branch). The resulting GO terms and their *P* values were used as input for REViGO^57^, a web tool to summarize lists of GO terms by removing redundant terms. To visualize the summarized GO terms associated with branch-dependent genes, we used the R package GOplot^58^. GOplot calculates a *z*-score for each GO term indicating if the term is more likely to be decreased (negative value) or increased (positive value): ${z{\mathrm{-score}}=\frac{{\mathrm{{up}-{down}}}}{\sqrt{{\mathrm{{count}}}}}}$, where ‘up’ and ‘down’ are the number of upregulated and downregulated genes, respectively, counting genes with log fold change > 0 between the U branch and L branch as upregulated genes.

### BATLAS

We used the web tool BATLAS^41^ to predict the percentage of brown adipocyte content in our data. We grouped cells from each Monocle developmental branch (progenitor, lower and upper) by pseudotime decile, to generate 30 groups of cells. The average expression for every gene in each decile was subsequently calculated on the normalized data in non-log space. The resulting matrix was used as input for BATLAS.

### Branch-specific gene expression analysis in pseudotime

Lists of human transcription factors^59^ and of the human secretome^28^ were used as input for identifying branch-specific gene expression over pseudotime. For each gene, smoothed gene expression across cells along pseudotime was determined, and the pseudotime point at which expression increases in one branch compared to the other was identified. To be considered as a valid result, we required that genes that were identified as diverging in expression between branches maintained the trend of divergence until end of pseudotime.

### Genetic prioritization analysis

We used CELLECT^60^ to genetically prioritize pseudo-temporal-ordered groups of cells. Specifically, we grouped cells from each Monocle developmental branch (progenitor, lower and upper) by pseudotime decile, to generate 30 groups of cells. We used CELLEX^60^ to calculate expression specificity of these cell groups. Briefly, CELLEX normalizes the expression data using a common transcript count (assuming 10,000 transcripts per cell) and log transformation is applied. Next, expression specificity likelihood (ES_μ_) is computed for groups of cells. We used CELLECT with S-LDSC as the genetic prioritization model. We ran CELLECT with default parameters (100-kb window size around each gene, correcting for baseline v1.1 and ‘all genes’ annotations). We performed CELLECT analysis for 39 GWAS traits.

### RNA-seq analysis

Analysis of bulk RNA-seq data is described in the R Notebook at https://github.com/cphbat/NatMetab2023_adipo_swat_singlecell/.

### Reference data integration and label transfer

Reference scRNA-seq and snRNA-seq datasets with annotations (labels) were obtained from Emont et al.^9^, Vijay et al.^44^ and Sun et al.^45^ and used for the label transfer process. References obtained in Seurat format were converted to anndata for use with the Scanpy framework^61^.

#### Scanorama integration

Each reference dataset was individually integrated with the scRNA-seq data generated in this paper (query data) using Scanorama through the Scanpy interface. After integration, a kNN classifier (from scikit-learn) was trained on the reference subset of the Scanorama joint embedding with the reference annotation labels as the target. Then, the query subset of the Scanorama joint embedding was used to predict the labels for the query cells. Confidence scores for the predicted labels were obtained from predict_proba function of the kNN classifier.

#### scNym annotation transfer

Each reference was individually concatenated with the query and the combined anndata were used for scNym^62^ analysis, with the query labels set to ‘unknown’. Default scNym training configuration was used except that learning rate was set to 0.1 and the training mode set to ‘new identity discovery’, to enable identification of out-of-sample cell types in the query data. Predicted query labels were exported for plotting with ggplot2. Training and prediction were performed on a GPU-enabled node (Nvidia Tesla V100) of Computerome cluster.

### Statistics and reproducibility

No statistical method was used to predetermine sample size. Sample size for the initial progenitor analysis (cells from at least three different donors for each of the four depots) was based on our previous experience with these cells^14–16^. Based on the observations within this article that progenitors across depots and donors were similar, we proceeded with cells from one donor for each depot when exploring the cells during early differentiation. The statistical analyses of the RNA-seq data were conducted using R or Python, and the statistical analysis of the oxygen consumption data and quantification of cell populations were performed with GraphPad Prism. For the oxygen consumption data in Fig. 5g, three data points were excluded. The reason is because, in these measurements, the drop after oligomycin injection was larger than expected (>50% compared to usually 20–25%). The experiments were not randomized, and the investigators were not blinded to allocation during experiments and outcome assessment. The staining performed in Figs. 3e, 4c and 5b and Extended Data Figs. 2a–f and 3 were reproduced with similar results at least once. The staining in Fig. 6a represents cells from ten individual clones that were expanded and differentiated separately.

### Reporting summary

Further information on research design is available in the Nature Portfolio Reporting Summary linked to this article.

## Supplementary information

Supplementary Information Supplementary Note and Supplementary Figs. 1–8

Reporting Summary

Supplementary Tables 1–14 Supplementary Table 1. Sample overview of preadipocytes. Supplementary Table 2. DESeq analysis between progenitors from different depots. Wilcoxon rank-sum test and multiple hypothesis testing using the Benjamini–Hochberg method was used. Supplementary Table 3. Sample overview time series. Supplementary Table 4. Number of P branch cells per depot. Supplementary Table 5. Branch-specific genes encoding secreted products. The analysis was based on BEAM analysis, using a likelihood ratio test to find branch-specific genes. Supplementary Table 6. Branch-specific transcription factors. The analysis was based on BEAM analysis, using a likelihood ratio test to find branch-specific genes. Supplementary Table 7. Genes clustered by BEAM analysis, using a likelihood ratio test to find branch-specific genes. Supplementary Table 8. Transcription factor list from the BEAM genes identified by a likelihood ratio test. Supplementary Table 9. REVIGO gene-set enrichment analysis on the BEAM output genes was done with the R package gProfileR. The *P* values are thus from gProfileR, which uses Fisher’s one-tailed test, also known as cumulative hypergeometric probability, as the *P* value measuring the randomness of the intersection between the query and the ontology term. The *P* value represents the probability of the observed intersection plus probabilities of all larger, extreme intersections. Supplementary Table 10. BATLAS results in pseudotime. Supplementary Table 11. GWAS for CELLECT analysis. Supplementary Table 12. CELLECT results. Supplementary Table 13. RNAscope probe list. Supplementary Table 14. Demuxlet results.

## Data Availability

RNA-seq data are deposited to the NCBI Gene Expression Omnibus under accession numbers GSE227635 (scRNA-seq data) and GSE223588 (bulk RNA-seq data). The human progenitor cells in the current study are non-immortalized cells derived from human biopsy samples, which can only be passaged a limited number of cycles and are therefore not available in scalable amounts for sharing.

## Code availability

The source code to reproduce all figures and tables is available at https://github.com/cphbat/NatMetab2023_adipo_swat_singlecell/. Code for Seahorse cell count is available at https://github.com/cphbat/Cell_count/.
