# Supplementary material for: Adipogenic and SWAT cells separate from a common progenitor in human brown and white adipose depots
Source: Nat Metab. 2023 Jun 19;5(6):996–1013. doi: 10.1038/s42255-023-00820-z (PMC10290958; doi:10.1038/s42255-023-00820-z)
Supplement: Supplementary file 1 — Supplementary Note and Supplementary Figs. 1–8 [file 42255_2023_820_MOESM1_ESM.pdf]

nature metabolism

Article https://doi.org/10.1038/s42255-023-00820-z

Adipogenic and SWAT cells separate from a common progenitor in human brown and white adipose depots

In the format provided by the authors and unedited

Supplementary note. Data exploration of progenitor single cell RNA sequencing data

To explore whether depot-dependent differences in single gene expression occurred, we first performed differential expression (DE) tests for each depot against the contrary two depots (e.g. cells from the two white depots were compared to cells from perirenal and then supraclavicular). We found 181 genes for supraclavicular, 129 for perirenal, 116 for subcutaneous and 108 for visceral (Bonferroni corrected $P < 0.05$, absolute average log fold change > 0.25). However, after inspecting these genes, we observed that most genes were found because of sample-specific signals. Therefore, we also performed DE tests for each sample against the contrary two depots and only kept the intersecting genes for all samples belonging to the same depot. This resulted in a shorter list of differentially expressed genes per depot: 6 genes for supraclavicular, 19 for perirenal, 11 for subcutaneous and 26 for visceral (Bonferroni corrected $P < 0.05$, absolute average log fold change > 0.25) (**Supplementary Table 2**). For the brown adipocyte progenitor samples, 3 genes stood out. The first gene, *TM4SF1*, was higher expressed in both the perirenal and supraclavicular depots (average log FC 0.87 and 0.34 respectively) and was found for all perirenal samples and 2 of the supraclavicular samples (**Figure S3**). *LY6K* was also higher expressed in the perirenal and supraclavicular samples (average log FC of 0.33 and 0.38 respectively) and was found for 2 perirenal samples and 2 supraclavicular samples (**Figure S3**). Last, *HOXB7* was found as a negative marker gene for all supraclavicular samples (average log FC -0.42, **Figure S3**). Furthermore, we found two genes that seemed to discriminate the visceral samples well. *BARX1* was found to be higher expressed in a small part of the cells in the visceral samples (average log FC 0.25, **Figure S3**), and *LINC01116* was found as a negative gene for all visceral samples (average log FC -0.32, **Figure S3**). We next explored the data using Monocle and Velocyto, which underscored the similarities between the adipogenic progenitor cells, assigning cell cycle effects as the major driver of differences (**Figure S4**).

Figure S1

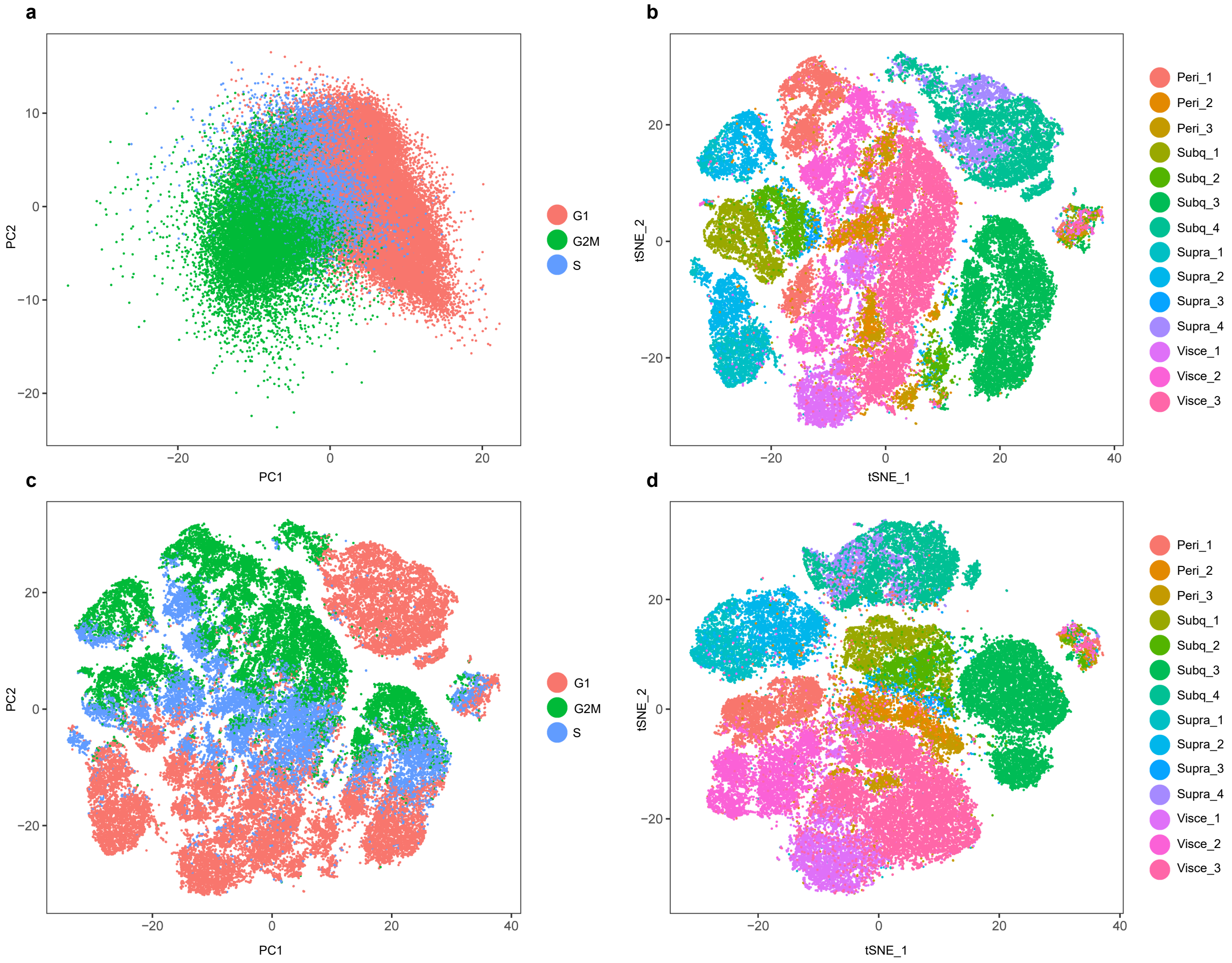

Figure S1. Visualizations of the preadipocyte dataset. a) Principal component analysis (PCA) plot, cells coloured on cell cycle phase. n=56,371 cells total, 12 samples; Peri 1, n=3,934; Peri 2, n=2,852, Peri 3, n=1,273, Subq 1, n=3,007, Subq 2, n=2,002, Subq 3, n=8,619, Subq 4, n=4,885, Supra 1, n=3,047, Supra 2, n=3,570, Supra 3, n=880, Supra 4, n=2,785, Visce 1, n=4,539, V isce 2, n=4,893, Visce 3, n=10,085. **b)** t-distributed stochastic neighbour embedding (t-SNE) plot of the first 15 principal components coloured on sample name and **c)** coloured on cell cycle phase. **d)** t-SNE plot of the first 12 principal components of the data with cell cycle effects regressed out coloured on sample name.

Figure S2

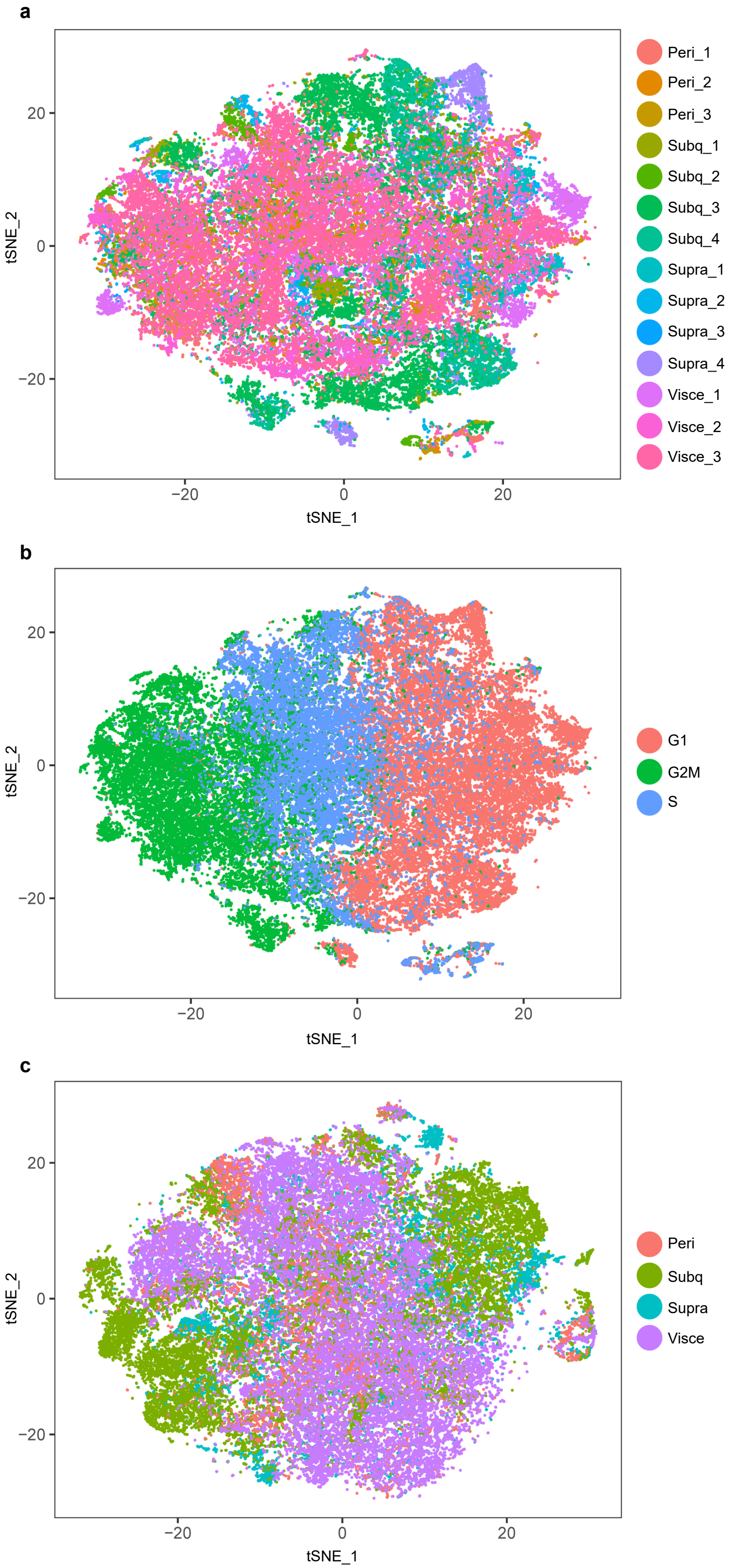

Figure S2. Alignment of the samples in the preadipocyte dataset. a) t-SNE plot of the first 15 aligned canonical components coloured on sample name and **b)** coloured on cell cycle phase. **c)** t-SNE plot of the first 15 aligned canonical components of the data with cell cycle effects regressed out.

Figure S3

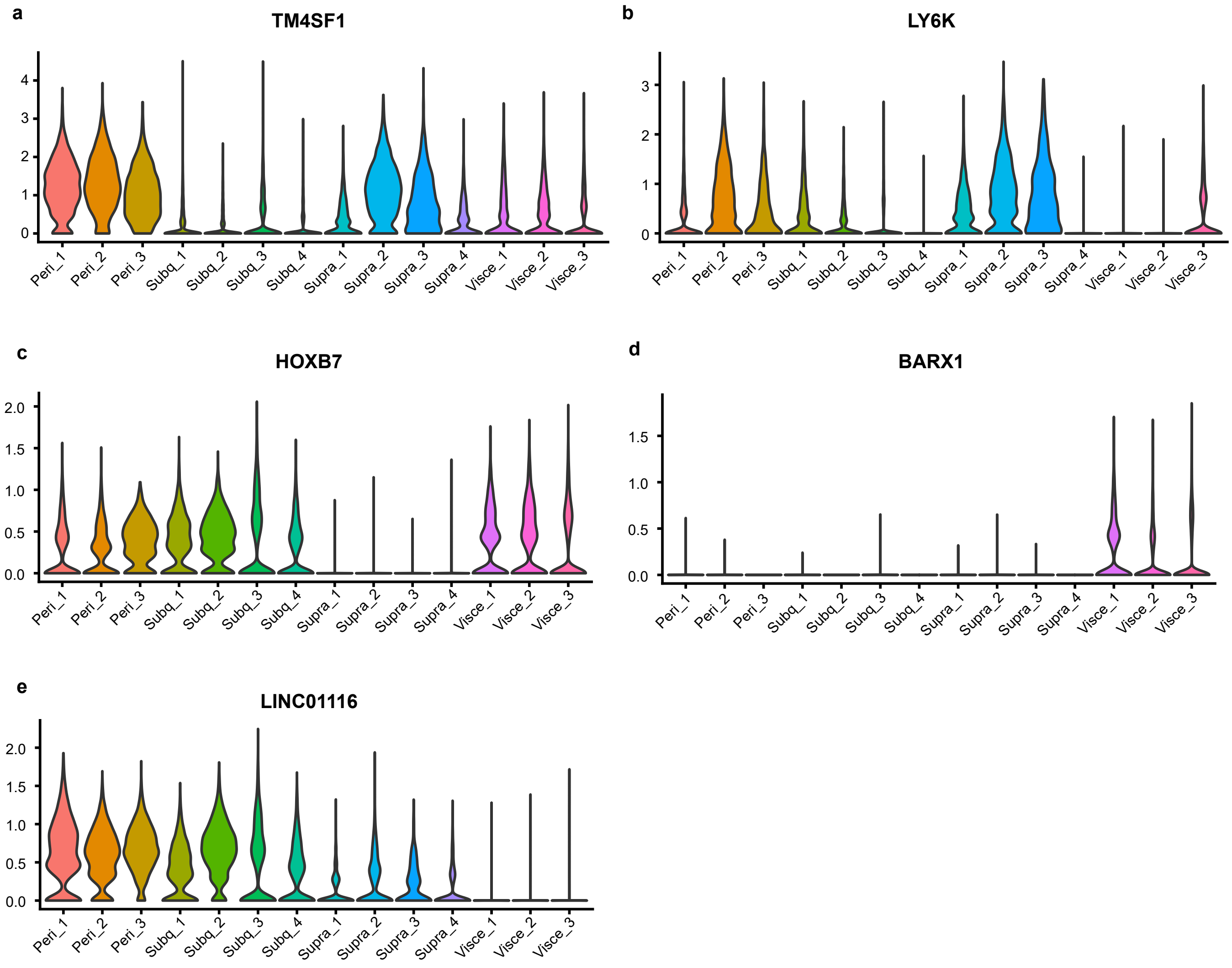

Figure S3. Violin plots of the expression of differentially expressed genes between depots in the preadipocyte dataset. Each depot was tested against the two depots of the contrary type. Y-axis shows the log normalized expression, X-axis the samples. **a)** TM4SF1 (avg log FC Peri = 0.87, Supra = 0.34). **b)** LY6K (avg log FC Peri = 0.33, Supra = 0.38). **c)** HOXB7 (avg log FC Supra = -0.42). **d)** BARX1 (avg log FC Visce = 0.25). **e)** LINC01116 (avg log FC Visce = -0.32)

Figure S4

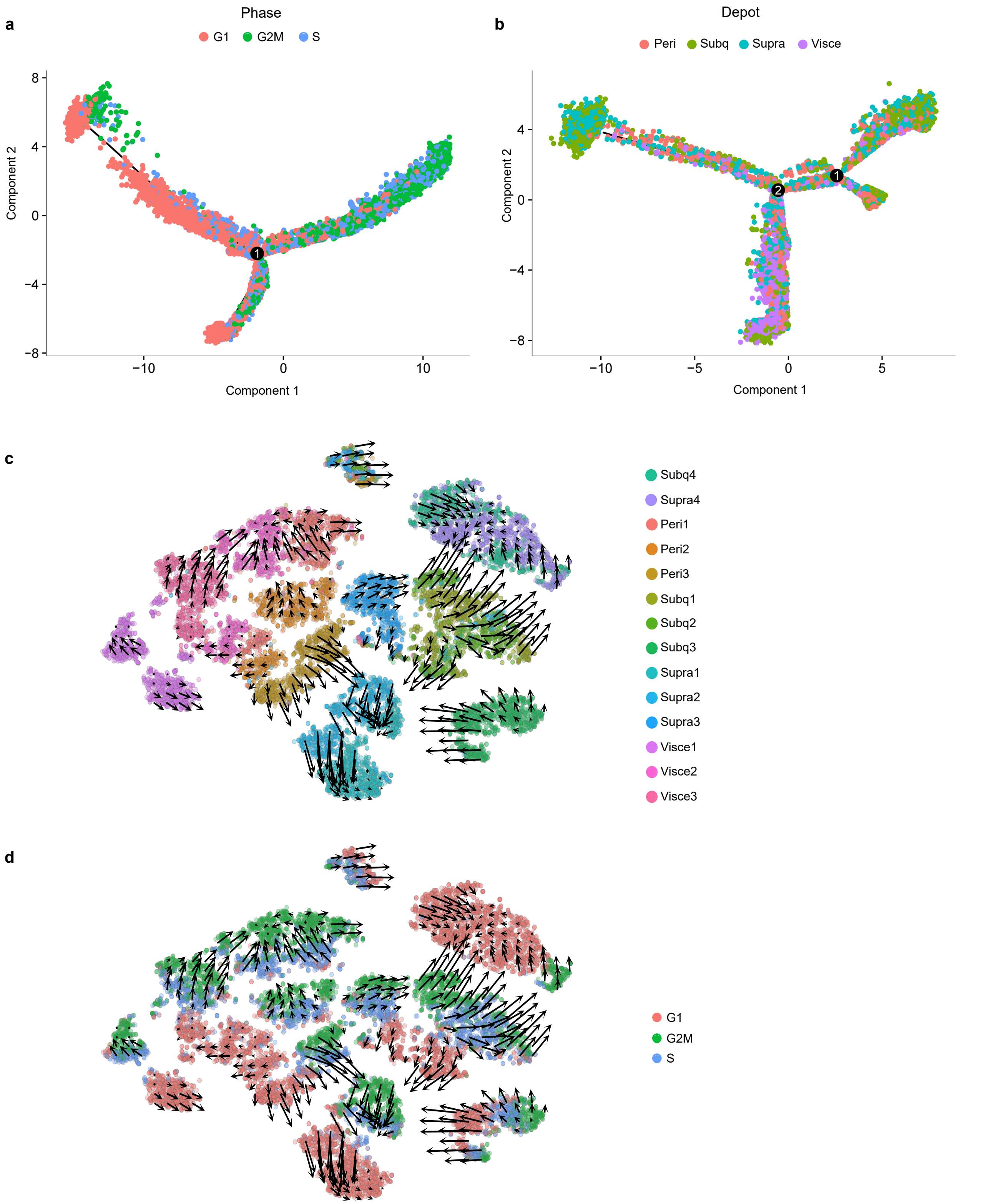

Figure S4. Cell development in the preadipocyte dataset. a) Cell developmental trajectory of the data (down sampled to 1,000 cells per sample) built with Monocle. The top 1,000 genes with the highest dispersion were used as features. Cells are coloured on assigned cell cycle phase. **b)** Developmental trajectory of the data (down sampled to 1,000 cells per sample) with cell cycle effects regressed out. **c)** t-SNE visualization of the preadipocyte dataset (down sampled to 1,000 cells per sample) coloured by sample name. The arrows represent the RNA velocity calculated with Velocyto and indicate the direction cells will go in gene expression space. **d)** t-SNE plot of the data with projected RNA velocity coloured on cell cycle phase.

Figure S5

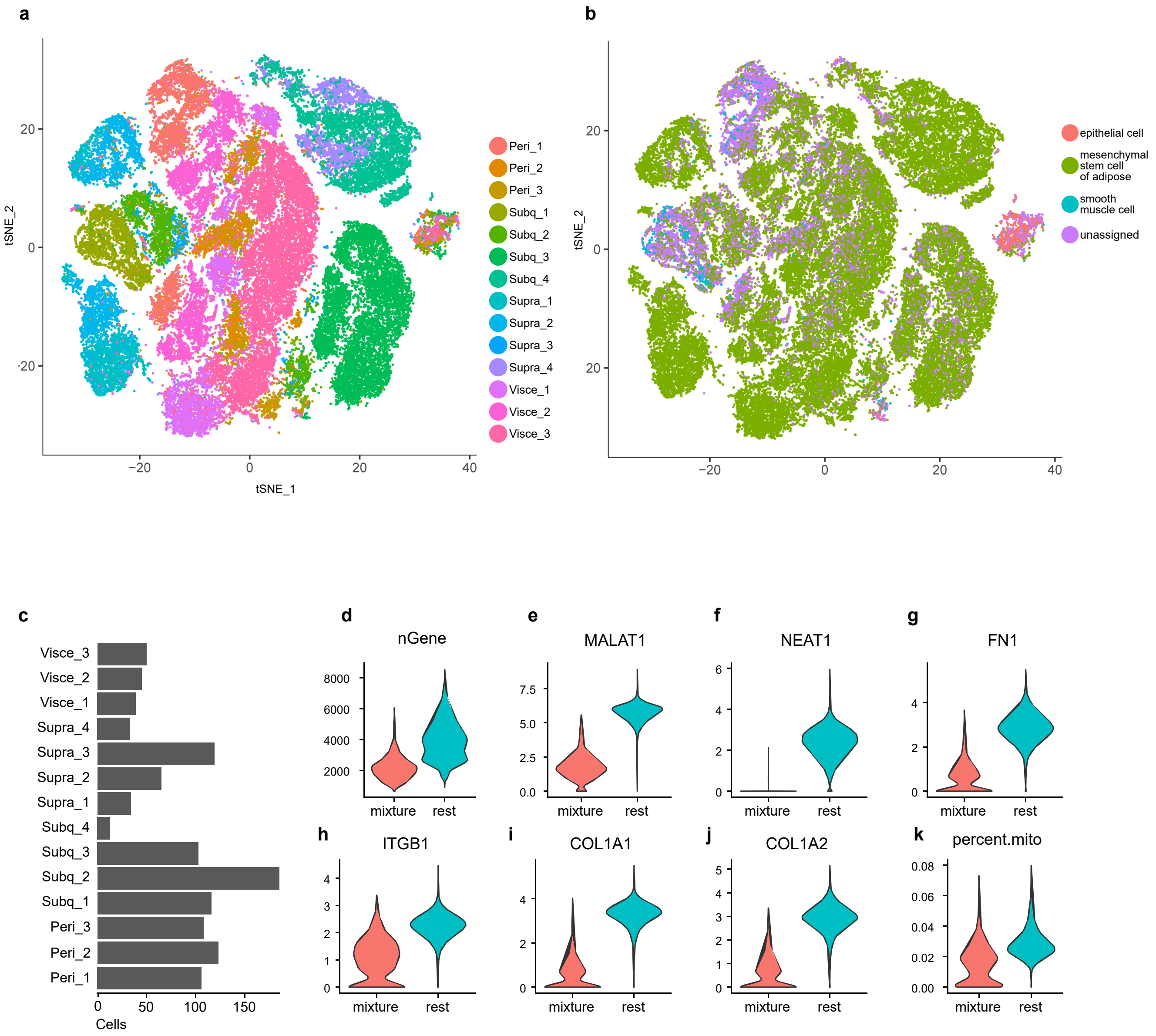

Figure S5. A subpopulation of CD29- cells ("common cluster"). a) t-SNE plot of the preadipocyte dataset with the subpopulation cluster highlighted. **b)** t-SNE plot of the preadipocyte dataset coloured on assigned cell type from the scmap analysis, using the Tabula Muris Fat dataset as a reference. **c)** Sample composition in the common cluster. **d)** Number of expressed genes in the common cluster and the rest of the cells in the preadipocyte dataset. **e-j)** Expression of MALAT1, NEAT1, FN1, ITGB (encodes for surface protein CD29), COL1A1 and COL1A2 in the common cluster (labelled as "mixture") and the rest of the cells. Y-axis shows the log-normalized expression. **k)** Percentage of mitochondria gene expression in the common cluster and the rest of the cells.

Figure S6

a

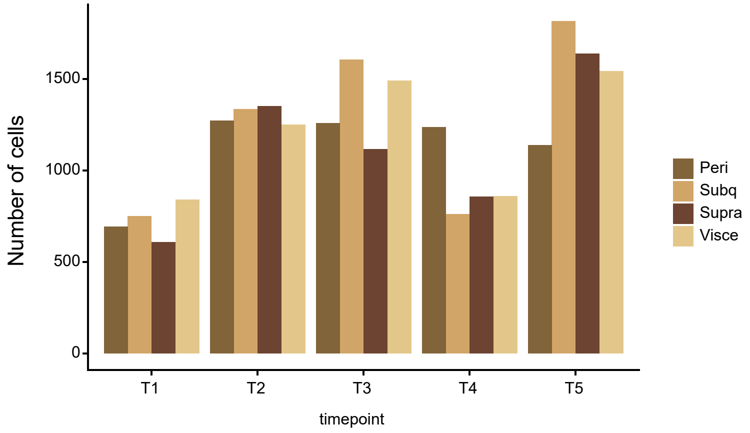

b

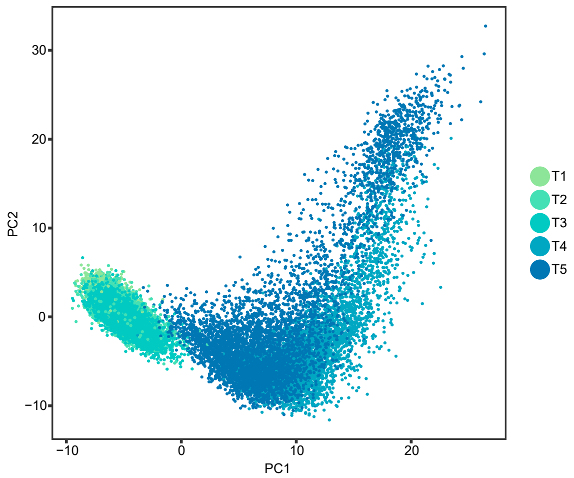

Figure S6. Data exploration of adipocytes at T1-T5 a) Number of cells per depot for each timepoint. **b)** Seurat alignment on timepoint and coloured by timepoint (left), depot (middle) and branch (right). **c)** PCA plot coloured on time point.

Figure S7

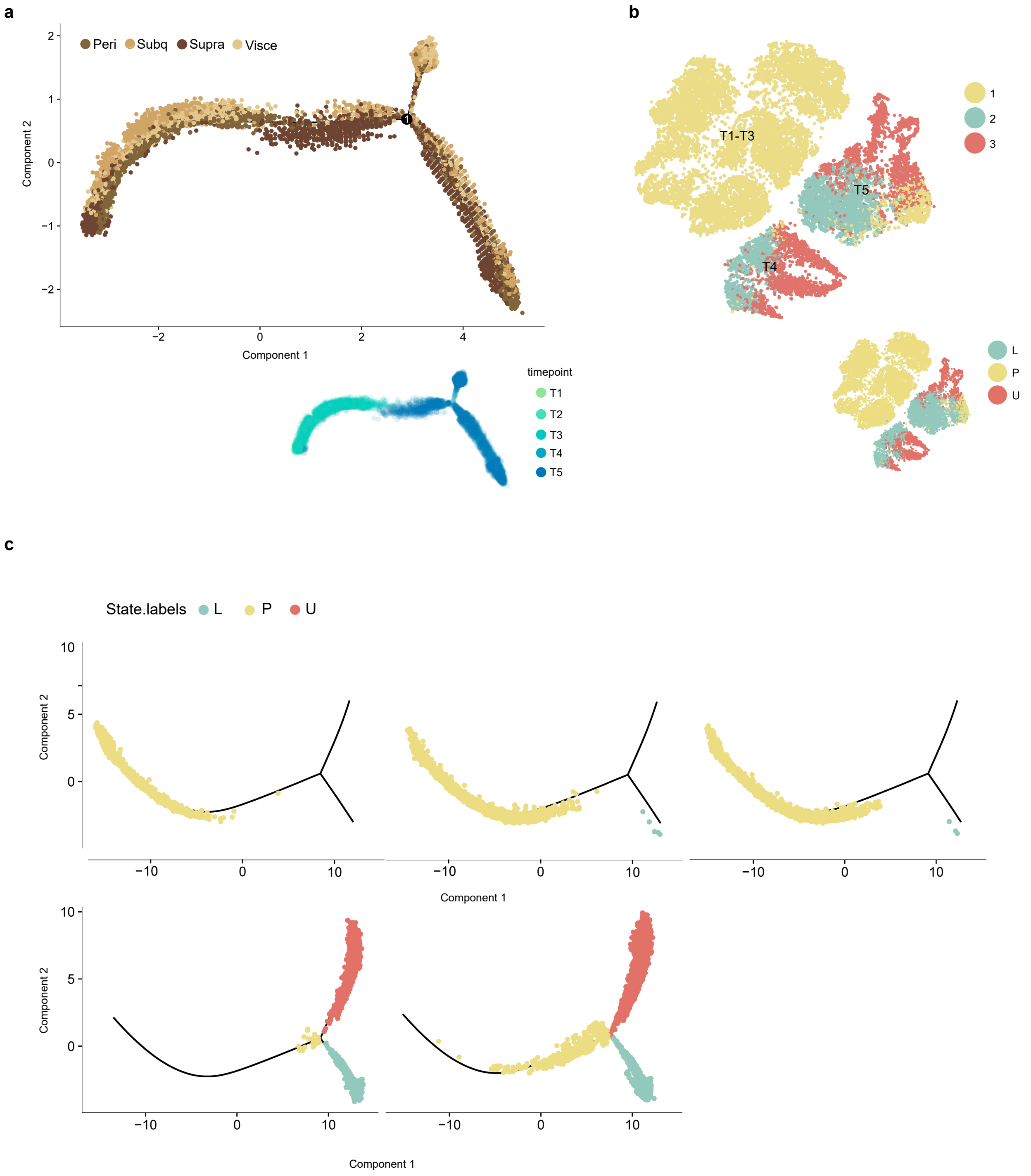

Figure S7. Monocle trajectory analysis. a) Monocle trajectory of the data built using DE genes between brown (perirenal and supraclavicular) and white (subcutaneous and visceral) as features. The negative binomial test was used for DE testing and results were filtered on absolute average logFC > 0.25, resulting in 82 DE genes between brown and white. Just as in the original trajectory, a branch split is identified separating the majority of brown and white. **b)** tSNE atlas of the data coloured on the new Monocle state, showing the new states are very similar to the original Monocle states shown in the inset. **c)** Monocle trajectories per timepoint coloured on branch. Cells from the first three timepoints form the P (Progenitor) branch. Cells in T4 and T5 split into the U (Upper) and L (Lower) branch, and both contain a subset of cells assigned to the P branch.

Figure S8

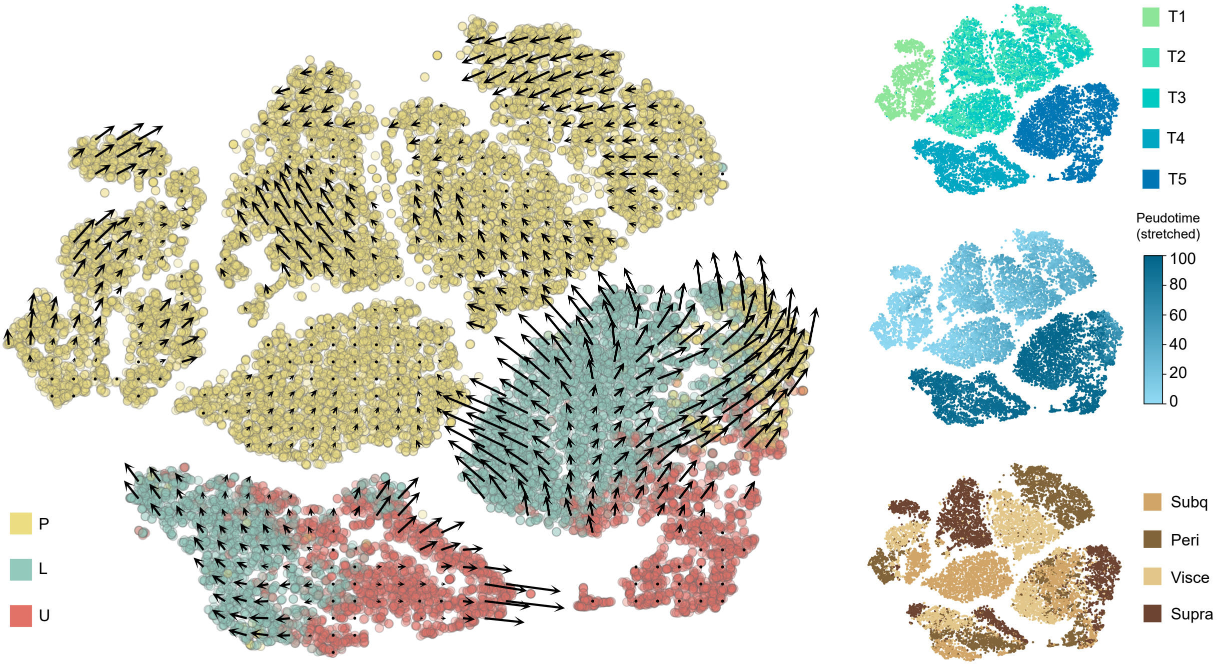

Figure S8. RNA Velocity confirms developmental trajectory analysis. Velocyto vector field overlaid on tSNE projection of the data. RNA velocity (arrows) indicates the average differentiation trajectories for cells located in different parts of the tSNE plot. RNA velocyto of cells from T4 and T5 confirms the developmental branch point inferred in the trajectory analysis. Insets on the right side show the same tSNE plot overlaid with time point, pseudotime and fat depot. The tSNE projection was obtained from the Velocyto workflow.
