# Supplementary material for: Adipogenic and SWAT cells separate from a common progenitor in human brown and white adipose depots
Source: Nat Metab. 2023 Jun 19;5(6):996–1013. doi: 10.1038/s42255-023-00820-z (PMC10290958; doi:10.1038/s42255-023-00820-z)
Supplement: Supplementary file 2 — Reporting Summary [file 42255_2023_820_MOESM2_ESM.pdf]

nature portfolio

Corresponding author(s): Søren Nielsen and Camilla Scheele

Last updated by author(s): Apr 13, 2023

Reporting Summary

Nature Portfolio wishes to improve the reproducibility of the work that we publish. This form provides structure for consistency and transparency in reporting. For further information on Nature Portfolio policies, see our Editorial Policies and the Editorial Policy Checklist.

Statistics

For all statistical analyses, confirm that the following items are present in the figure legend, table legend, main text, or Methods section.

n/a	Confirmed	
☐	☒	The exact sample size (*n*) for each experimental group/condition, given as a discrete number and unit of measurement
☐	☒	A statement on whether measurements were taken from distinct samples or whether the same sample was measured repeatedly
☐	☒	The statistical test(s) used AND whether they are one- or two-sided<br>*Only common tests should be described solely by name; describe more complex techniques in the Methods section.*
☒	☐	A description of all covariates tested
☐	☒	A description of any assumptions or corrections, such as tests of normality and adjustment for multiple comparisons
☐	☒	A full description of the statistical parameters including central tendency (e.g. means) or other basic estimates (e.g. regression coefficient) AND variation (e.g. standard deviation) or associated estimates of uncertainty (e.g. confidence intervals)
☐	☒	For null hypothesis testing, the test statistic (e.g. *F*, *t*, *r*) with confidence intervals, effect sizes, degrees of freedom and *P* value noted<br>*Give P values as exact values whenever suitable.*
☒	☐	For Bayesian analysis, information on the choice of priors and Markov chain Monte Carlo settings
☒	☐	For hierarchical and complex designs, identification of the appropriate level for tests and full reporting of outcomes
☒	☐	Estimates of effect sizes (e.g. Cohen's *d*, Pearson's *r*), indicating how they were calculated

Our web collection on statistics for biologists contains articles on many of the points above.

Software and code

Policy information about availability of computer code

Data collection	Single-cell transcritomics: lOX Genomics Cell Ranger v. 2.0.1; Genotyping: Illumina GenomeStudio v2.0; Single-cell demultiplexing: Demuxlet v1.0; Image acquisition: Evos FL (Life Technologies), Leica Thunder, BioTek Cytation 1 with Gen5 V3.11 software, Molecular Devices Image Xpress Pico automated Cell Imaging; Extracellular flux analysis Agilent Seahorse 96XFe analyzer with Wave 2.6.3 software; FACS sorting for cloning experiment Biosciences BD FACS Melody Cell Sorter.
Data analysis	The source code to reproduce all figures and tables for this manuscript are available at https://github.com/cphbat/NatMetab2023_adipo_swat_singlecell . Seurat v. 2.3.4 for all Seurat analysis except for Seurat Integration analysis where v. 3.1.0 was used, Monocle (v. 2.8.0), BATLAS, REVIGO, GOplot. Scanorama, scNym, scikit-learn. Bulk RNA-seq - Salmon 1.5.2 & DESeq2. Leica LASX software and ImageJ V were used for image adjustments. Cell Reporter Xpress analysis software was used for quantification of cell types based on FISH staining. GraphPad Prism V9.3. was used to analyze in vitro experiments. Microsoft Excel 2016 was used to calculate Seahorse data. The Python 3.10.6 source code for quantification of nuclei for seahorse data normalization is available at https://github.com/cphbat/Cell_count.

For manuscripts utilizing custom algorithms or software that are central to the research but not yet described in published literature, software must be made available to editors and reviewers. We strongly encourage code deposition in a community repository (e.g. GitHub). See the Nature Portfolio guidelines for submitting code & software for further information.

Data

Policy information about availability of data

All manuscripts must include a data availability statement. This statement should provide the following information, where applicable:

- Accession codes, unique identifiers, or web links for publicly available datasets
- A description of any restrictions on data availability
- For clinical datasets or third party data, please ensure that the statement adheres to our policy

Raw and processed scRNA-seq & Bulk RNA-seq data are publicly accessible from NCBI under the accessions GSE227635 & GSE223588

Human research participants

Policy information about studies involving human research participants and Sex and Gender in Research.

Reporting on sex and gender	Female participants
Population characteristics	Preadipocyte samples (Time point T1)<br>Supraclavicular samples: BMI 23 (21-24), Age 51 (48-62)<br>Perirenal samples : BMI 25 (22-28), Age 54 (44-60)<br>Subcutaneous samples: BMI 22 (20-24), Age 53 (47-62)<br>Visceral samples: BMI <25, Age 25 (29-41)<br><br>Dfferentiation time series (Time points T1-T5): Age 51 (40-60), BMI 23 (21-24)
Recruitment	The progenitor cells are assembled from patients undergoing gallbladder surgery (visceral adipose progenitors), nephrectomy surgery (perirenal adipose progenitors) or during surgery in patients with suspected cancer in the neck area (supraclavicular adipose progenitors) or with the Bergström needle biopsy method (subcutaneous adipose progenitors). The subjects provided written and informed consent and the studies were performed in accordance with the Helsinki declaration.
Ethics oversight	The Scientific Ethics Committee of the Capital Region of Denmark

Note that full information on the approval of the study protocol must also be provided in the manuscript.

Field-specific reporting

Please select the one below that is the best fit for your research. If you are not sure, read the appropriate sections before making your selection.

☒ Life sciences ☐ Behavioural & social sciences ☐ Ecological, evolutionary & environmental sciences

For a reference copy of the document with all sections, see nature.com/documents/nr-reporting-summary-flat.pdf

Life sciences study design

All studies must disclose on these points even when the disclosure is negative.

Sample size	No power calculation was performed for the single cell experiments, we included adipocytes from four different human adipose depots, at different time points of differentiation, where cells from all depots were pooled for each time point to avoid batch effect. As we were looking for common or differential populations between cells from different origins, we concluded that on culture from each depot was sufficient. For in vitro experiments, no sample size calculations were performed. Sample size was determined based on studies using comparable methods ands cells in our lab or reported in the literature. n-numbers are indicated as n=X biologically independent cells in the figure legends.
Data exclusions	We used the Demuxlet algorithm which allows for genetic deconvolution of sample identity and doublet detection in single-cell libraries with samples pooled across individuals. We discarded 3,444 cells (12.8 % of total) identified by Demuxlet as doublets.<br>Seahorse experiment: We routinely observe an initial drop in baseline OCR measurements due to adaptation. Therefore, the 1st basal measurements were excluded based on our previous experience. In Figure 5g (stimulated proton leak), three values were excluded to due issues with oligomycin measurements/injections leading to >50% OCR drop compared to other wells (20-25% drop). In Figure 4e-f, 3 single Rotenone/Antimycin measurments were excluded due to negative values, which cannot be possible (PMCID: PMC5298256). These exclusions are based on our routine observations but not according to clear pre-established cut-offs.
Replication	We started out with comparing n=3 different cultures from each of the four depots, of cells at proliferating state. The lack of clustering was replicated with the proliferating state from the second experiment (were additional time points during differentiation also was performed). The finding of two different cell types was confirmed by FISH microscopy, which we have reproduced in >3 separate experiments. All attempts of replication were successful. Bulk RNA-Sequencing data on enriched SWAT cells cultured in proliferation or differentiation media were

generated from 4 independent separation experiments with cells from the same donor. Enrichments of cell populations were performed at least three times. All enrichments were successful. In vitro experiments with enriched cell populations were performed once with brown adipose progenitor cells from one donor separated into at least three individual cultures. Given the heterogeneity within this cell population and the potential variations in enrichment purity reflected between wells, these cultures should be considered individual samples rather than technical replicates.

Randomization	This is not relevant for the current study as it was a characterization of cell types without treatment.
Blinding	Blinding was not relevant as the analysis was of explorative nature.

Reporting for specific materials, systems and methods

We require information from authors about some types of materials, experimental systems and methods used in many studies. Here, indicate whether each material, system or method listed is relevant to your study. If you are not sure if a list item applies to your research, read the appropriate section before selecting a response.

Materials & experimental systems

n/a	Involved in the study	
☐	☒	Antibodies
☐	☒	Eukaryotic cell lines
☒	☐	Palaeontology and archaeology
☒	☐	Animals and other organisms
☒	☐	Clinical data
☒	☐	Dual use research of concern

Methods

n/a	Involved in the study	
☒	☐	ChIP-seq
☒	☐	Flow cytometry
☒	☐	MRI-based neuroimaging

Antibodies

Antibodies used	Anti-perilipin monoclonal antibody, rabbit IgG, Science Signaling, Clone D1D8, Cat. number 9349<br>Alex Fluor 488 conjugated donkey anti-rabbit IgG (H+L) highly cross-adsorbed secondary antibody (Invitrogen, A-21206)
Validation	Perilipin antibody: validation by IF on mouse brown adipose tissue sections and differentiated 3T3-L1 adipocytes. Perilipin staining only demonstrated in lipid-containing adipocytes. Validation reference for IF on human Transverse carpal ligament sections to detect lipomatosis (fat deposition): PMID: 32686688<br>Alex Fluor 488 conjugated donkey anti-rabbit IgG: Validation according to manufacturer's data sheet.

Eukaryotic cell lines

Policy information about cell lines and Sex and Gender in Research

Cell line source(s)	Progenitor cells were isolated from perirenal, supraclavicular, subcutaneous and visceral human adipose tissues.
Authentication	The cell cultures were treated separately from isolation and throughout differentiation and until harvest in our lab.
Mycoplasma contamination	All cell cultures tested negative for mycoplasma contamination.
Commonly misidentified lines<br>(See ICLAC register)	Not applicable.
